# Photosystem I-independent oxygenic photosynthesis in cyanobacteria

**DOI:** 10.1038/s41467-026-74903-2

**Published:** 2026-07-10

**Authors:** Marta Ludwiczak, Marcel Dann, Theo Figueroa-Gonzalez, Eslam M. Abdel-Salam, Weiyang Chen, Serena Schwenkert, Martin Lehmann, Milena Zhivkovikj, Maysoon Noureddine, Markéta Linhartová, Sadanand Gupta, Josef Komenda, Arthur Guljamov, Stefania Viola, Feng Liu, Dario Leister

**Affiliations:** 1 Plant Molecular Biology, Faculty of Biology, Ludwig-Maximilians-University Munich, Munich, Germany; 2 Mass Spectrometry of Biomolecules Service Unit at LMU (MSBioLMU), Faculty of Biology, Ludwig-Maximilians University Munich, Munich, Germany; 3 Institute of Microbiology of the Czech Academy of Sciences, Centre Algatech, Třeboň, Czech Republic; 4 Faculty of Science, University of South Bohemia, České Budějovice, Czech Republic; 5 Present Address: Bio-Inspired Energy Conversion, Technical University of Darmstadt, Darmstadt, Germany; 6 Present Address: Center for Electromicrobiology, Department of Biology, Aarhus University, Aarhus, Denmark; 7 Present Address: Microbiology, Department of Microbiology, Institute of Biochemistry and Biology, University of Potsdam, Potsdam, Germany; 8 Present Address: Photosynthesis & Environment (P&E) Team, CEA, CNRS, BIAM, UMR7265, Saint-Paul-Lez-Durance, France

**Keywords:** Photosystem I, Molecular evolution, Cellular microbiology

## Abstract

Oxygenic photosynthesis generates ATP and NADPH via linear electron flow from water to NADP^+^, a process thought to require photosystem I (PSI) for reductant formation. Here we demonstrate that oxygenic photosynthesis can operate without PSI in the cyanobacterium *Synechocystis* sp. PCC 6803. Using genetic engineering and adaptive laboratory evolution, we obtained PSI-deficient lineages capable of photoautotrophic growth, inorganic carbon fixation, and light-driven oxygen evolution. PSI-independent photoautotrophy arose from co-mutations in at least two proteins, including the translation elongation factor G (FusA), and required a functional NDH-1 complex. We propose that the light-driven electron transport in the evolved strains is reorganised in two branches: one involving terminal oxidases to generate proton motive force, and a second in which reverse NDH-1 activity exploits this gradient to produce reductant. These findings uncover unexpected plasticity in the thylakoid electron transport network and prompt a reassessment of the canonical requirement for PSI in oxygenic photosynthesis.

## Abstract (web summary)

Ludwiczak *et al*. show that cyanobacteria can grow and fix carbon dioxide through oxygenic photosynthesis without Photosystem I. Adaptive mutations enable an alternative electron transport pathway that replaces this previously essential complex.

## Introduction

**Fig. 1:**
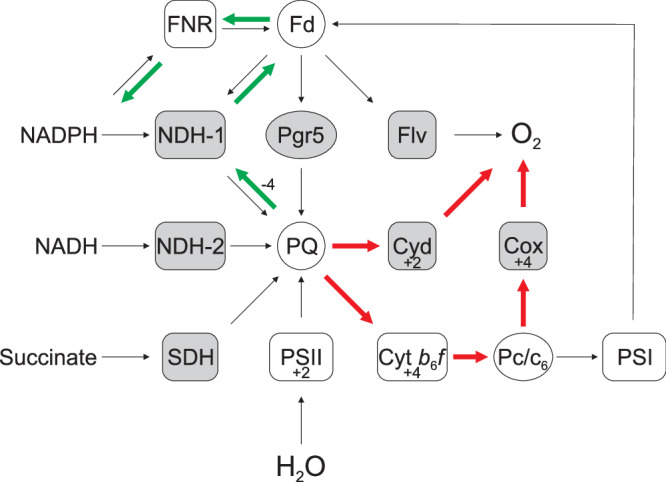
Thylakoid electron transport circuits in *Synechocystis* sp.PCC 6803. Components of linear electron flow (LEF) are displayed on a white background, whereas cyclic (CEF) and pseudocyclic (PCEF) pathways are shaded grey. Thick green arrows indicate the proposed NADPH-generating, pmf-consuming route mediated by reverse electron transfer activity of NDH-1, while red arrows represent the pmf-generating electron flow involving oxidases (see main text), potentially supporting the photoautotrophic growth of strains lacking PSI. Positive and negative numbers denote the number of protons contributing or reducing pmf. Abbreviations: Cyt *b*_6_*f* Cytochrome *b*_6_*f*; c_6_ Cytochrome c_6_, Cox Cyt *c* oxidase; Cyd Cyt *bd* quinol oxidase, Fd ferredoxin; Flv flavodiiron protein; FNR Fd-NADP^+^ reductase; NDH-1 NAD(P)H dehydrogenase-like complex 1, NDH-2 Type II NAD(P)H dehydrogenase; Pc plastocyanin; Pgr5 proton gradient regulation 5, PQ plastoquinone; PSI/II Photosystem I/II; SDH succinate dehydrogenase.

Photosynthetic electron transport in cyanobacteria, algae, and plants operates through a complex interplay of linear (LEF), cyclic (CEF), and pseudocyclic (PCEF) electron flows, each adjusted in a species-specific manner to balance energy demands and adapt to stress. In cyanobacteria such as *Synechocystis* sp. PCC 6803 (hereafter *Synechocysti*s), photosynthetic and respiratory electron transport chains are integrated within the thylakoid membrane, creating a highly versatile redox network supported by shared electron carriers, including plastoquinone (PQ) and the cytochrome (Cyt) *b*_6_*f* complex^1,2^ (Fig. 1).

LEF, conserved in all oxygenic phototrophs, transports electrons from water at photosystem II (PSII) via the PQ pool, the Cyt *b*_6_*f* complex, and plastocyanin (Pc) or Cyt *c*_6_, to photosystem I (PSI), where NADPH is produced^3^ (Fig. 1). Concurrently, the proton gradient formed during water oxidation and electron transfer through Cyt *b*_6_*f* drives ATP synthesis. Beyond this canonical pathway, electron transport modes diverge. CEF around PSI enhances ATP production without net NADPH production via PGR5-dependent processes, prevalent in C_3_ plants and algae, and the NDH-1 complex, especially in cyanobacteria and C_4_ plants^4–8^. Both PGR5 and NDH-1 pathways are thought to return electrons from ferredoxin (Fd) to the PQ pool^9–13^. In parallel, PCEF offers alternative electron sinks through reactions such as the Mehler (water-water) cycle, in which PSI reduces O_2_ to superoxide that is subsequently detoxified, and via flavodiiron proteins (Flvs), which directly reduce O_2_ to H_2_O^4^. Terminal oxidases similarly divert electrons from PQ or Pc/Cyt *c*_6_ to O_2_, mitigating the build-up of excess reducing equivalents under stress.

Oxygenic photosynthesis fundamentally depends on the coordinated function of PSII and PSI acting in series^3^; deletion of PSI typically abolishes photoautotrophic growth. Some algal and cyanobacterial strains lacking PSI can, however, survive heterotrophically on glucose or acetate^14–17^. In *Synechocystis*, the removal of PSI in combination with the terminal oxidase Cox, or the inactivation of Cyt *b*_6_ *f*, is lethal even under heterotrophic conditions, as no viable mutants can be obtained^18–21^. This lethality likely reflects the fatal over-reduction of the PQ pool^18^.

Here we show that oxygenic photosynthesis can persist in the absence of PSI. Through adaptive laboratory evolution (ALE) of a PSI-deficient *Synechocystis* strain, we obtained photoautotrophic lineages carrying adaptive point mutations, including recurrent substitutions in *slr1463* (*fusA*), encoding one of three elongation factor G proteins in *Synechocystis*. ALE of a ∆*psaAB* strain harbouring these *fusA* variants similarly restored photoautotrophy. Characterisation of these PSI-less photoautotrophic strains indicates that NDH-1 can substitute for PSI by operating in reverse, transferring electrons from PQ to Fd.

## Results

### Evolving photoautotrophy in PSI-deficient strains

**Fig. 2:**
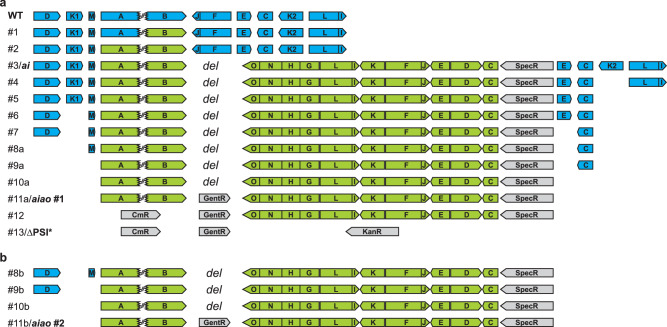
Generation of PSI-less *Synechocystis* strains. **a** Stepwise replacement of native *Synechocystis* PSI genes (blue) with *Arabidopsis thaliana* PSI subunit genes (green). The “*ai*” strain (“all-in”) carries all 14 plant and 8 cyanobacterial PSI genes, whereas “*aiao*” (“all-in, all-out”) retains only the plant set. The *psaA/B* operon is not drawn to scale. A 4.7 kb deletion (“*del*”) introduced in step 3 was repaired in step 11 resulting in insertion of a gentamicin-resistance marker (GentR). In steps 12-13, At*psaA/B* and At*psaONHGLIKFJEDC* operons were replaced by chloramphenicol- and kanamycin-resistance markers (CmR, KanR), respectively, generating a PSI-null strain (ΔPSI*). **b** Alternative construction route for *aiao* #2. This pathway differs from that of *aiao* #1 by the sequential order in which the *Synechocystis* genes encoding PsaC, PsaD and PsaM were deleted.

As part of our effort to replace the endogenous PSI of *Synechocystis* sp. PCC 6803 with the PSI complex from *Arabidopsis thaliana*^22^, we constructed a strain lacking all 12 *Synechocystis* PSI subunit genes (*psaA–psaF*, *psaI*, *psaJ*, *psaK1*, *psaK2*, *psaL*, and *psaM*) while introducing the 14 corresponding *A. thaliana* genes (*PsaA–PsaL*, *PsaN*, and *PsaO*) (Fig. 2, see “Methods”).

**Fig. 3:**
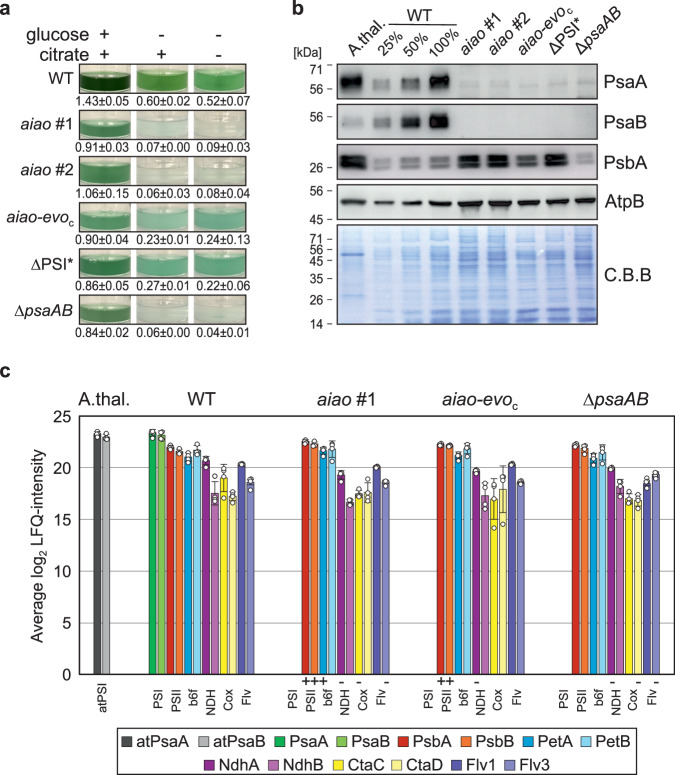
Photoautotrophic growth of PSI-deficient *Synechocystis* strains. **a** Growth under mixotrophic conditions (5 mM glucose) or photoautotrophic conditions (lacking glucose, with or without citrate) was assessed after 10 days at 30 °C in BG11 medium. Optical density at 720 nm (OD_720nm_) is shown (mean ± SD, *n* = 3). Cultures were inoculated at an OD_720nm_ of 0.1; values below 0.1 after 10 days indicate loss of viability. **b** Immunoblot analysis confirms the absence of the PSI core proteins PsaA and PsaB proteins in *aiao* and derived strains. **c** LC-MS-based proteomic analysis reveals accumulation of PSII (PsbA, PsbB), cytochrome *b*_6_*f* (PetA, PetB), NDH (NdhA, NdhB), cytochrome c oxidase (CtaC, CtaD) and flavodiiron proteins (Flv1, Flv3), while PSI core subunits (PsaA, PsaB) are not detected. Data represent mean ± SD (*n* = 4). Significant genotype-dependent changes relative to wild type (WT) are indicated by “+” (increase) and “-“ (decrease) (see Methods). Controls: WT, *A. thaliana* (A.thal), and Δ*psaAB*.

During the third step of this replacement process, a 4.7 kb deletion upstream of *psaF* - encompassing *sll1217*, which encodes a PGRL1-like protein that functions in CEF in concert together with Pgr5^23^, was introduced inadvertently and subsequently restored in step 11 (Fig. 2). After the seventh step of the replacement process, two independent transformation routes yielded strains with identical PSI gene composition (Fig. 2). These strains were designated “*aiao* #1” and “*aiao* #2” (“all plant genes in, all bacterial genes out”). Both strains exhibited strictly heterotrophic growth, proliferating only in the presence of glucose (Fig. 3a). Immunoblotting and LC–MS/MS-based proteomic analyses confirmed the absence of both cyanobacterial and plant PSI core subunits (Fig. 3b, c; Source Data). This finding suggests that the introduced plant PSI subunits are either insufficiently expressed or fail to assemble properly in *Synechocystis*, potentially due to the absence of plant-specific assembly factors that cannot be functionally replaced by endogenous components.

In contrast, other thylakoid protein complexes and flavodiiron proteins accumulated in the PSI-deficient strains (Fig. 3c). Notably, PSII core proteins were present at increased abundance in the *aiao* strains and their derivatives (Fig. 3b, c), suggesting compensatory remodelling of the photosynthetic apparatus in response to the loss of PSI.

To promote adaptive accumulation or assembly of plant PSI proteins, we subjected 12 replicate cultures (six per parental strain, *aiao* #1 and *aiao* #2) to photoautotrophic growth conditions under low light and without glucose. After 12–19 weeks, eight of these cultures initiated slow but sustained growth (Supplementary Fig. 1; Fig. 3a). The resulting strains, referred to as “*aiao-evo*” (*aiao-evo*_a_ to *aiao-evo*_h_), retained typical PSII and Cyt *b*_6_ *f* components but contained no detectable PSI subunits from either *Synechocystis* or *A. thaliana* (Fig. 3b, c). This was unexpected, as oxygenic photoautotrophy is generally thought to require coordinated PSII and PSI function, and earlier claims of PSI-independent photosynthesis in *Chlamydomonas reinhardtii* have been refuted^17,24^.

To confirm that the observed growth reflected true oxygenic photoautotrophy, we first excluded the possibility of alternative carbon utilisation. The *aiao-evo* strains maintained photoautotrophic growth in carbon-depleted medium, ruling out the use of trace organics such as citrate (Fig. 3a). We then removed all *A. thaliana* PSI genes from *aiao-evo*_c_ by homologous recombination, generating a ΔPSI* strain completely devoid of cyanobacterial or plant PSI loci (Fig. 2 and Fig. 3b, c). Remarkably, this strain retained photoautotrophic growth (Fig. 3a), establishing that *Synechocystis* can, under certain conditions, perform oxygenic photosynthesis in the absence of PSI.

### Adaptive mutations underlying photoautotrophy

Whole-genome resequencing of the eight *aiao-evo* lines, their two *aiao* progenitors, and the wild type (WT) identified 496 total sequence variants relative to the *Synechocystis* Kazusa reference genome^25^ (Source Data, Supplementary Tables 1 and 2). After excluding silent mutations, fully segregated WT variants, and pseudogene changes, 151 unique mutations remained in the *aiao* and *aiao-evo* lineages. Twenty mutations across 17 genes were specific to the *aiao* strains, while additional variants shared by *aiao* and *aiao-evo* cultures likely arose during strain construction (*pre-evolution*; Supplementary Table 3). Notably, each of the four replicate sets of *aiao-evo* cultures possessed unique combinations of 35–42 mutations, providing candidates linked to the acquisition of photoautotrophy.

Seven of the eight photoautotrophic cultures harboured nonsynonymous mutations in *fusA* (*slr1463*), encoding elongation factor G (EF-G), previously implicated in oxidative stress tolerance and PSII repair^26,27^. Nine distinct *fusA* alleles were detected, with *fusA*_*I28T*_ (three independent occurrences) and *fusA*_*P307L*_ (four occurrences) being most frequent (Table 1). The remaining culture did not exhibit *fusA* mutations but contained a segregated mutation in *lmbP* (*slr0862*). Notably, the same *lmbP* variant appeared in three additional *aiao-evo* populations. The *lmbP* gene encodes a member of the GHMP kinase superfamily, a group of structurally related enzymes that catalyse ATP-dependent phosphorylation of small metabolites and play essential roles in pathways such as amino acid biosynthesis, carbohydrate metabolism, and the mevalonate pathway^28,29^. Indeed, network interaction analyses^30^ show LmbP clustering with metabolic enzymes, including a phosphoheptose isomerase and other proteins involved in central metabolism, supporting its proposed function in sugar metabolism or carbohydrate processing. Accordingly, it has been annotated as a “probable sugar kinase” in *Synechocystis* sp. LKSZ1.

**Table 1:** Adaptive mutations unique to *aiao-evo* strains

Batch	Gene	Codon Mutation	Amino acid change	Frequency of mutation [%]	Sequencing depth
*aiao*-*evo*_a_	*fusA*	ATT → ACT	I28T	18.90	168
		ATT → ACT	I132T	7.90	175
		CCC → CTC	P307L	73.10	150
	*lmbP*	GCG → ACG	A95T	7.59	174
*aiao-evo*_b_	*lmbP*	GCG → GTG	A95V	100.00	157
*aiao-evo*_c_	*fusA*	GAT → AAT	D98N	7.80	121
		CCC → CTC	P307L	13.10	140
	*lmbP*	GCG → ACG	A95T	10.00	140
		GCG → GTG	A95V	62.89	140
*aiao-evo*_d_	*fusA*	ATT → ACT	I28T	45.60	131
		ATT → ACT	I132T	18.60	156
		CCC → CTC	P307L	40.80	166
*aiao*-*evo*_e_	*fusA*	ATC → ACC	I375T	88.40	144
		72 bp tandem duplication	+ 24 aa extension at C-terminus	5.70	131
*aiao*-*evo*_f_	*fusA*	CCC → CTC	P254L	6.50	133
		ATC → ACC	I375T	94.60	132
		72 bp tandem duplication	+24 aa extension at C-terminus	5.50	142
*aiao*-*evo*_g_	*fusA*	ATT → ACT	I28T	52.60	131
		CCC → CTC	P307L	41.40	125
*aiao*-*evo*_h_	*fusA*	TCC → CCC	S366P	8.90	191
		CGT → CAT	R624H	72.90	174
	*lmbP*	GTT → TTT	V75F	6.23	215
		GCG → ACG	A95T	19.28	201

Candidate genes potentially conferring photoautotrophic capacity were identified based on two criteria: (i) absence of mutations in the WT or *pre-evolution aiao* control, and (ii) presence of at least two independent mutant alleles among *aiao-evo* batches, with at least one allele reaching a frequency >20%. The independently evolved lineages were derived from the two separate *aiao* progenitors: *aiao* #1 (*aiao-evo*_a_ to *aiao-evo*_d_) and *aiao* #2 (*aiao-evo*_e_ to *aiao-evo*_h_). The ‘Frequency of mutation’ column indicates the percentage of reads carrying the mutant allele at that position. The ‘Sequencing depth’ column reports the total number of sequencing reads covering the respective mutation site.

**Fig. 4:**
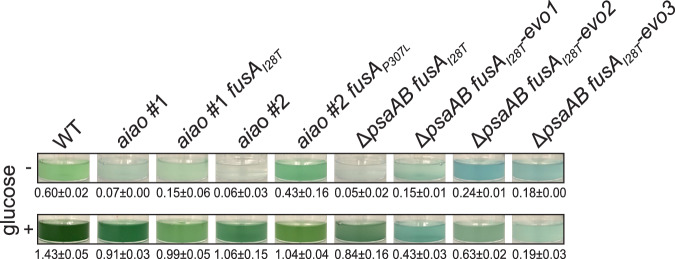
Adaptive mutations confer photoautotrophy in PSI-deficient *Synechocystis.* After inoculation to an OD_720nm_ of 0.1, strains were cultured for 10 days at 30 °C in BG11 medium under mixotrophic (5 mM glucose) or autotrophic (no glucose) conditions and 3 µmol photons m^−2^ s^−1^. Optical density at 720 nm (OD_720nm_) is shown (mean ± SD, *n* = 3). Cultures were inoculated at an OD_720nm_ of 0.1; values below 0.1 after 10 days indicate loss of viability. Non‑autotrophic *aiao* #1 and *aiao* #2 regained photoautotrophy upon introduction of *fusA*_*I28T*_ and *fusA*_*P307L*_ mutations, respectively. Expression of *fusA*_*I28T*_ in the Δ*psaAB* background did not restore autotrophy, but adaptive evolution of this strain yielded three independent Δ*psaAB fusA*_*I28T*_*‑evo* lines with autotrophic growth. These results show that *fusA* mutations, combined with additional adaptive changes, enable photoautotrophy in the absence of PSI.

To test causality, we introduced *fusA*_*I28T*_ and *fusA*_*P307L*_ into the parental *aiao* #1 and *aiao* #2 strains, respectively. Both engineered strains regained photoautotrophic growth (Fig. 4), demonstrating that single amino acid substitutions in *fusA* can confer photoautotrophy in a PSI-deficient background. However, introducing *fusA*_*I28T*_ into a freshly constructed Δ*psaAB* strain did not restore photoautotrophy (Fig. 4), indicating that additional adaptive mutations acquired during the generation of the *aiao* strains were also required.

To identify these secondary changes, we initiated adaptive laboratory evolution (ALE) experiments using Δ*psaAB fusA*_*I28T*_ as a starting point (see Methods). After prolonged selection, three out of more than one hundred independent cultures achieved photoautotrophic growth (designated Δ*psaAB fusA*_*I28T*_-*evo*1–3; Fig. 4). Control ALE experiments using Δ*psaAB* without the *fusA* mutation were unsuccessful, suggesting that *fusA* substitutions act as a prerequisite for the rare combination of additional adaptive events that enable PSI-independent photoautotrophy, explaining the absence of such phenotypes in decades of PSI deletion studies.

### Pigment composition and light tolerance in the absence of PSI

**Fig. 5:**
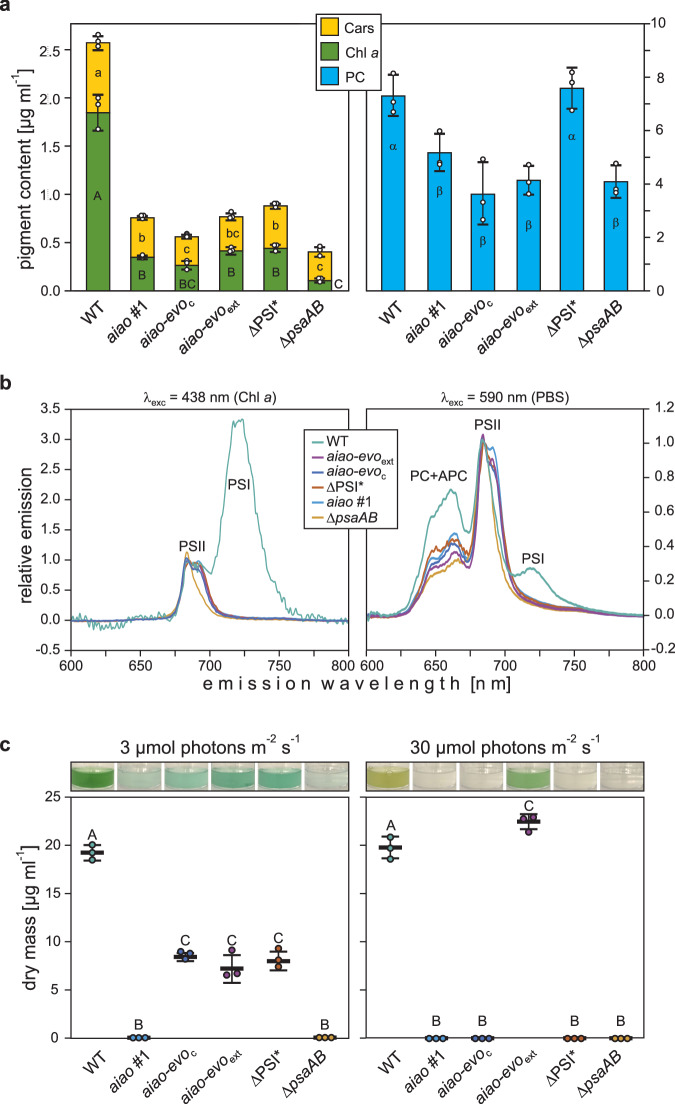
Pigment composition and light tolerance in the absence of PSI. **a** Pigment composition of WT and PSI-deficient strains grown mixotrophically (5 mM glucose, 30 °C, 3 µmol photons m^−2^ s^−1^). Chlorophyll *a* (Chl *a*), carotenoids (Cars), and phycocyanin (PC) were quantified (see Methods). Average values ± SDs (error bars) are provided. Statistical differences (*p* ≤ 0.05, *post-hoc* Bonferroni-Holm HSD) for *n* = 3 biological replicates after significant among-group differences have been found by two-sided one-way ANOVA (Chl *a*: *p*  =  2.20 × 10^−11^, Cars: *p*  =  6.70 × 10^−8^, PC: *p*  =  8.61 × 10^−5^) are indicated by letters. **b** 77 K fluorescence emission spectra of samples from autotrophic (WT, *aiao-evo*_*c*_, *aiao-evo*_*ext*_, ΔPSI^*^) and mixotrophic (*aiao* #1, Δ*psaAB*) cultures (3 µmol photons m^−2^ s^−1^), excited at 438 nm (Chl *a*) or 590 nm (phycobilisomes). Spectra were normalised to the PSII F685 nm peak (*n* ≥ 3). Emission bands: 640-670 nm (phycocyanin + allophycocyanin), 685-695 nm (PSII Chl *a*), 715-730 nm (PSI Chl *a*). **c** Photoautotrophic growth (no glucose) visualised after 10 days and quantified as dry mass following cultivation at 3 and 30 µmol photons m^−2^ s^−1^. Average values ± SDs (error bars) are provided. Significant differences (*p* ≤ 0.05, *post-hoc* Bonferroni-Holm HSD) for *n* = 3 biological replicates after significant among-group differences have been found by two-sided one-way ANOVA (3 µmol photons m^−2^ s^−1^: *p*  =  1.46 × 10^−11^, 30 µmol photons m^−2^ s^−1^: *p*  =  8.88 × 10^−16^) are indicated by letters.

Because PSI (notably PsaA and PsaB) binds the majority of cellular chlorophyll *a* (Chl *a*)^31,32^, PSI depletion in these mutants caused a pronounced reduction in total Chl *a* relative to WT cells, consistent with previous reports^16^ (Fig. 5a, Source Data). Carotenoid and phycocyanin (PC) levels also declined but to a lesser extent, and PC content in the photoautotrophic ΔPSI* strain was comparable to WT (Fig. 5a).

Low-temperature (77 K) fluorescence spectroscopy confirmed the absence of PSI emission features in all *aiao* derivatives, whereas PSII-originating peaks persisted (Fig. 5b, Source Data), demonstrating intact energy transfer from antenna pigments to PSII reaction centres.

Photoautotrophic PSI-less strains were highly light-sensitive, growing only under very low illumination (3 µmol photons m^−2^ s^−1^), reaching an average of 44 ± 2% (*aiao-evo*_c_), 37 ± 7% (*aiao-evo*_ext_), and 42 ± 5% (ΔPSI*) of WT dry mass per ml of culture, while bleaching at intensities tenfold higher (Fig. 5c, Source Data), a known characteristic of PSI depletion^33^. Prolonged laboratory evolution gradually improved light tolerance, leading to an “extended evolution” variant, *aiao-evo*_ext_ (that is derived from *aiao-evo*_*c*_), which grew comparably to WT at 30 µmol photons m^−2 ^s^−1^ (Fig. 5c).

### Photosynthetic and respiratory activities in the absence of PSI

**Fig. 6:**
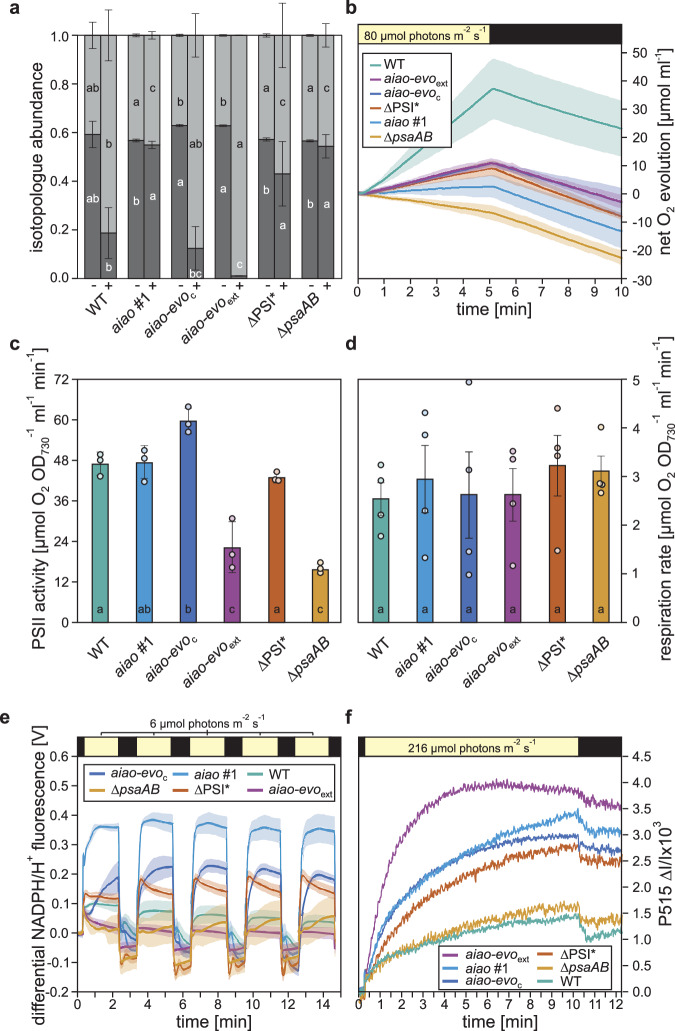
Photosynthetic and respiratory activities in the absence of PSI. **a** Inorganic carbon fixation assessed by ^13^C incorporation into sucrose. Feeding with ^13^C bicarbonate (+) shifted isotopomer abundance towards heavier forms (light grey) relative to the monoisotopologue (dark grey) in photoautotrophic (WT, aiao*-evo*_*c*_, *aiao-evo*_*ext*_ and ΔPSI*) but not in non-photoautotrophic strains (*aiao* #1 and Δ*psaAB*). Feeding with bicarbonate with natural isotope composition (-) served as control. Average values ± SDs (error bars) are provided. Differences between treatment were evaluated by ANOVA and Duncan’s test for *n* = 3 independent cultures per strain. **b** Light-dependent net oxygen evolution in cultures of OD_720nm_ = 1.2 (5-min light pulse, 80 µmol photons m^−2^ s^−1^), followed by darkness. Traces represent mean values (lines) ± SE (shaded areas) of *n* = 4 biological replicates. **c** PSII activity was determined by measuring steady-state oxygen evolution in the presence of 0.5 mM DCBQ and 1 mM K_3_Fe(CN)_6_ at 80 µmol photons m^−2^ s^−1^. Measurements were performed on culture samples adjusted to OD_720nm_ = 2.0 (*n* = 3 biological replicates). Data were normalised to OD_720nm_ = 1. Average values ± SDs (error bars) are provided. **d** Dark respiration rates after 5-min illumination with 80 µmol photons m^−2^ s^−1^. Columns represent mean values ± SE of *n* = 4 biological replicates. Individual data points are indicated. **e** Light-dependent NADPH generation and consumption in the dark. Traces represent mean values (lines) ± SD (shaded areas) from *n* = 3 biological replicates. **f** The *aiao* #1 strain and its derivatives show increased P515 signal accumulation under red actinic light. P515 signals were recorded from dark-incubated ( > 16 h) *Synechocystis* cells of the indicated genotypes during illumination with red actinic light (216 μmol photons m^−2^ s^−1^, λ  =  635 nm). The timing of actinic light onset and offset is indicated by the bar at the top of the panel. Traces represent mean values (lines) ± SD (shaded areas) from *n* = 6/4/8/4/4/4 biological replicates of WT/*aiao*#1/*aiao-evo*_*c*_/*aiao-evo*_*ext*_/ΔPSI*/Δ*psaAB*, respectively. P515 signals are expressed as ΔI/I × 10^3^ of the 550–520 nm difference signal. For a/c/d, letters indicate significant differences (*p*  ≤  0.05; two-sided one-way ANOVA, *p*  =  9.43 × 10^−3^ (a, -), *p*  =  6.55 × 10^−6^ (a, +), *p*  =  1.84 × 10^−7^ (c) and *p* = 0.95 (d); a, *post-hoc* Duncan’s test; c-d, *post-hoc* Bonferroni-Holm-corrected Tukey HSD.

CO_2_ fixation capacity was assessed via incorporation of ^13^C from labelled bicarbonate into sucrose, a Calvin–Benson cycle metabolite, monitored by GC–MS (Fig. 6a, Supplementary Fig. 2, Source Data). The *aiao-evo*_c,_ *aiao-evo*_ext_ and ΔPSI* strains showed measurable ^13^C enrichment, comparable to WT, confirming their ability to fix inorganic carbon. In contrast, the *aiao* #1 and Δ*psaAB* strains did not display significant labelling.

Oxygen evolution assays further corroborated oxygenic photosynthesis: illumination increased net O_2_ release in all photoautotrophic PSI-less strains, though at lower rates than WT, reaching an average of 29 ± 2% (*aiao-evo*_c_), 29 ± 6% (*aiao-evo*_*ext*_), and 24 ± 16% (ΔPSI*) of WT net O_2_ evolution rates (Fig. 6b, Source Data). Consistent with earlier studies^17,33^, Δ*psaAB* itself retained residual light-dependent O_2_ evolution (when compared to the dark), while the parental *aiao* #1 strain exhibited a modest increase in O_2_ production, correlated with enhanced PSII protein and Chl *a* accumulation (Figs. 3b, c and 5a). This indicates that the adaptive mutations present in *aiao* #1 are already positively affecting PSII activity, either directly or by enhancing light tolerance in general. In fact, direct measurements of PSII activity, based on O_2_ evolution in the presence of DCBQ and K_3_Fe(CN)_6_, revealed higher values in the *aiao* #1 strain relative to Δ*psaAB*. Meanwhile, PSI-deficient photoautrophic strains retained WT-like PSII activity, with the exception of *aiao-evo*_ext_, which showed reduced rates (Fig. 6c). Respiration rates, calculated from the data underlying Fig. 6b, were comparable across all genotypes (Fig. 6 d).

The kinetics of light-induced NADPH formation and its subsequent dark consumption were assessed in vivo by monitoring NADPH fluorescence^34,35^. Upon illumination, the amplitude of the NADPH signal was elevated in the *aiao* #1, *aiao-evo*_c_ and ΔPSI* strains relative to WT and Δ*psaAB* (Fig. 6e), indicating enhanced NADPH formation, a reduced or delayed rate of NADPH-consuming reactions in the light, or a more oxidised NADPH pool in the dark. In contrast, the *aiao-evo*_ext_ strain exhibited a lower NADPH signal amplitude.

Measurements of the proton motive force (pmf) using the non-invasive P515 method^36^ revealed that the PSI-deficient, non-autotrophic *aiao* #1 strain and its evolved autotrophic derivatives, most prominently *aiao-evo*_ext_, displayed accelerated light-induced pmf generation and higher steady-state pmf levels under actinic illumination compared to WT and Δ*psaAB* (Fig. 6 f).

Taken together with PSII activity and NADPH dynamics, these findings indicate that adaptation to higher light intensities in the *aiao-evo*_ext_ strain is associated with substantial reconfiguration of cellular electron and proton fluxes.

### Potential role of NDH-1 in PSI-independent photoautotrophy

Under conditions of elevated pmf, as observed in the PSI-deficient *aiao* #1 strain and its evolved autotrophic derivatives, the NDH-1 complex has been suggested to operate in reverse, mediating electron transfer from plastoquinone (PQ) to ferredoxin (Fd) while simultaneously dissipating the proton gradient across the thylakoid membrane^11,37,38^. The overall abundance of the NDH-1 complex was only marginally influenced by the absence of PSI. Specifically, NdhA levels were slightly reduced in *aiao* #1, *aiao-evo*_c_, and Δ*psaAB* compared to WT, whereas NdhB accumulation remained unchanged (see Fig. 3c).

**Fig. 7:**
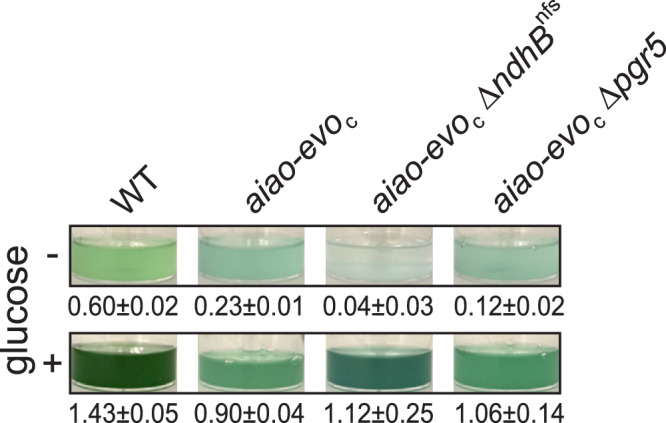
NDH-1, but not Pgr5, is essential for PSI-independent photoautotrophy. Growth of *aiao‑evo*_c_ Δ*ndhBⁿᶠˢ* (“not fully segregated”) and *aiao‑evo*_c_ Δ*pgr5* mutants under autotrophic (no glucose) or mixotrophic (5 mM glucose) conditions at 30 °C and 3 µmol photons m^−2^ s^−1^. Optical density at 720 nm (OD_720nm_) is shown (mean ± SD, *n* = 3). Cultures were inoculated at an OD_720nm_ of 0.1; values below 0.1 after 10 days indicate loss of viability. Disruption of *ndhB* abolished photoautotrophy, whereas *pgr5* deletion permitted autotrophic growth but reduced growth rate, demonstrating that NDH‑1, but not Pgr5, is required for PSI‑independent photosynthesis.

We next disrupted the *ndhB* gene, essential for NDH‑1 assembly^39,40^ but dispensable for photoautotrophic growth in a WT background^40–43^, in the *aiao-evo*_c_ strain. Complete segregation of the deletion could not be achieved (Supplementary Fig. 3), and the resulting partially segregated *aiao-evo*_c_ Δ*ndhB*^*nfs*^ strain (“nfs” standing for “not fully segregated”) failed to grow photoautotrophically. In contrast, deletion of *pgr5*, affecting a parallel cyclic electron flow route, did not impair photoautotrophic growth (Fig. 7).

These findings indicate that NDH‑1 activity might be indispensable for PSI-independent photoautotrophy, possibly by substituting PSI’s electron transfer function in NADPH generation following the acquisition of key adaptive mutations. Alternatively, NDH-1 may not directly contribute to NADPH generation; instead, the combined effect of PSI and NDH-1 deficiency could be more pronounced than that of PSI and Pgr5 deficiency.

### Proton stoichiometries for potential electron pathways in PSI-less strains

To assess whether PSI‑independent NADPH production can sustain carbon fixation, we quantified the proton production and translocation associated with electron flow through the photosynthetic and respiratory chains, using established stoichiometries, normalised per two electrons (2 e⁻) (Table 2, Fig. 1). During canonical LEF, water oxidation at PSII contributes +2 H^+^ to the lumen while the Cyt *b*_6_*f* complex transfers an additional +4 H^+^ via the Q-cycle, making a total of +6 H^+^ per 2 e⁻. In PSI-deficient strains, NADPH might be formed via reverse electron flow through NDH-1 instead, which consumes 4 H⁺ per 2 e⁻ to drive NADP^+^ reduction. Coupled solely to PSII, the PSI-independent pathway H_2_O → PQ → NDH-1 → NADPH therefore yields a net −2 H^+^ per 2 e⁻ ( + 2 from PSII, −4 from NDH-1). This potential NADPH-producing branch depletes pmf and cannot independently sustain ATP synthesis.

**Table 2:** Net proton translocation associated with distinct electron-transfer pathways

Pathway	PSII	Cyt *b*_6_*f*	NDH-1	Cyd	Cox	Net H^+^
H_2_O → NADP^+^ (LEF)	+2	+4	-	-	-	+6
CEF	-	+4	+4	-	-	+8
H_2_O → NADP^+^ (rev NDH-1)	+2	-	−4	-	-	−2
H_2_O → O_2_ (Cyd)	+2	-	-	+2	-	+4
H_2_O → O_2_ (Cox)	+2	+4	-		+4	+10

Stoichiometries are normalised to two transferred electrons (2 e⁻). Total lumenal proton release (ΔH⁺) includes contributions from PSII water oxidation (+ 2H^+^), the cytochrome *b*_6_*f* Q-cycle (+ 4 H^+^) and, where applicable, the oxidases Cyd (+ 2H^+^) and Cox (+ 4 H^+^). During CEF, electron flow through NDH-1 produces 4 H⁺ per 2 e⁻, but consumes 4 H⁺ per 2 e⁻ for NADP^+^ reduction in the reverse mode. Net positive values indicate pmf generation (lumenal acidification), while negative values indicate pmf consumption. LEF, linear electron flow; Cyt *b*_6_*f*, Cytochrome *b*_6_*f*; Cyd, *bd*-type quinol oxidase; Cox, aa_3_-type cytochrome.

Respiratory oxidases could provide the compensatory proton source. Oxygen reduction via the bd-type quinol oxidase (Cyd) contributes +2 H^+^ per 2 e^-^, while the aa_3_-type cytochrome c oxidase (Cox) contributes +4 H^+^ per 2 e^-^. Consequently, the PSII + Cyd pathway (Cyd-only) yields +4 H⁺/2 e^-^, and the PSII + Cyt *b*_6_*f* + Cox route (Cox-only) yields +10 H⁺/2 e^-^. Assuming equal flux through Cyd and Cox, the mixed oxidase regime produces +7 H⁺ per 2 e⁻. Thus, in PSI-deficient conditions, oxidase activity might constitute the pmf-generating branch.

### Electron partitioning required to meet Calvin-Benson cycle demand

To fulfil the ATP/NADPH requirement of 1.5 imposed by the Calvin–Benson cycle, we modelled the distribution of electron flux between reverse NDH-1 activity and terminal oxidases (Table 3, Methods). Under mixed Cyd+Cox activity, ~56% of electrons must be directed to the oxidases, generating 3.07 H⁺ per 2 e⁻ and ~0.66 ATP/2 e⁻. In a Cyd-only scenario, ~69% of electrons are channelled through Cyd, yielding 2.15 H⁺ per 2 e^-^ and ~0.46 ATP/2 e⁻. By contrast, in a Cox-only configuration, ~47% of electrons pass via Cox, producing 3.69 H^+^ per 2 e^-^ and ~0.79 ATP per 2 e⁻. Thus, at a fixed Calvin–Benson ATP/NADPH ratio, the Cox-only mode generates the highest pmf and ATP yield per 2 e^−^, while sustaining slightly more than 50% of the net oxygen production observed in WT cells operating LEF and CEF with PSI. However, if an elevated pmf were to inhibit Cyt *b*_6_*f* activity^44^, thereby limiting electron supply to Cox and shifting O_2_ reduction predominantly to Cyd, net O_2_ evolution would decline to approximately 30% of WT levels (Table 3).

**Table 3:** Electron partitioning, ATP and NADPH production, and net oxygen evolution under distinct electron-transfer regimes

Wild type (PSI present)						
Regime (WT)	LEF fraction	CEF fraction	NADPH	ΔH⁺	ATP	Net O_2_
LEF + CEF	0.875	0.125	1.000	7.00	1.50	0.50

PSI-less strains (NDH-1_reverse_ + oxidase respiration)						
Oxidase regime	*x* (NDH-1_reverse_)	Oxidase fraction (1 - *x*)	NADPH	ΔH⁺	ATP	Net O_2_
Cyd-only	0.308	0.692	0.308	2.15	0.46	0.154
Cyd+Cox (1:1)	0.437	0.563	0.437	3.07	0.66	0.219
Cox-only	0.526	0.474	0.526	3.69	0.79	0.263

All values are normalised to two electrons extracted from water (2 e⁻), corresponding to the evolution of 0.5 O_2_ at PSII. Proton translocation (ΔH^+^) and ATP yields are calculated assuming an ATP synthase stoichiometry of 4.67 H^+^ per ATP. For PSI-less strains, *x* denotes the fraction of electrons allocated to reverse NDH-1 for NADP^+^ reduction, while (1 − *x*) is directed to terminal oxidases. Net O_2_ evolution is calculated as 0.5*x*, reflecting the balance between O_2_ released at PSII and O_2_ consumed by oxidases. Electron partitioning was solved to satisfy the Calvin–Benson cycle requirement (ATP/NADPH = 1.5). For comparison, wild-type (WT) values are shown for a LEF/CEF distribution of 0.875/0.125, which restores the required ATP/NADPH ratio.

## Discussion

Our results demonstrate that photoautotrophic growth in *Synechocystis* does not strictly dependent on PSI, challenging the conventional understanding of photosynthetic electron transport. The data are consistent with a model in which LEF could be functionally substituted by two routes diverging at the PQ pool, one supplying reductant and the other driving pmf. While this dual‑pathway scenario remains hypothetical, it highlights the remarkable plasticity of the thylakoid network, where photosynthetic and respiratory components coexist and cooperate within a shared membrane system. Such flexibility could enable partial uncoupling of ATP and NADPH generation, offering both conceptual insights and applied potential.

A distinct combination of adaptive mutations enables photoautotrophic growth in the absence of PSI. The necessity for concurrent mutations in *fusA* or *lmbP*, together with alterations in at least one additional locus, likely explains why PSI-independent photoautotrophy has not been identified despite extensive investigations of PSI-deficient *Synechocystis* strains over the past three decades^14–16,33,45,46^. The extended construction and maintenance of our *aiao* lines under low-light conditions likely facilitated the spontaneous emergence of one of these critical mutations. Supporting this interpretation, even non-evolved *aiao* mutants exhibit an enhanced light-induced proton motive force (pmf) formation signature and WT-like PSII activity, whereas a newly generated Δ*psaAB* mutant displays P515 kinetics similar to those of the parental WT and very little PSII activity (Fig. 6c, f). In addition, the transient deletion of *sll1217*, a component of CEF^23^, may have triggered compensatory adaptations influencing NDH-1 activity or alternative PSI-bypass pathways.

Disruption of NDH‑1 abolished photoautotrophic growth in the evolved strains, suggesting that the PSI-deficient strains are either operating at the very edge of photosynthetic viability - where additional perturbations such as loss of NDH-1 become lethal - or that NDH-1 functionally compensates for the absence of PSI. The elevated pmf levels observed in *aiao* #1 and its photoautotrophic derivatives (see Fig. 6) are consistent with NDH‑1 operating in reverse, transferring electrons from PQ to Fd. This reverse electron flow is energetically feasible, driven by the proton motive force (pmf), and resembles the uphill electron transfer mediated by complex I in anoxygenic phototrophs, such as purple non-sulphur bacteria, where electrons are transferred from ubiquinol to NAD⁺^47–49^. Comparable reverse activity has been proposed for the chloroplast NDH-1 complex under conditions of elevated pmf ( ~ 200 mV) and a highly reduced PQ pool^11^. In *Synechocystis*, the redox properties of NDH‑1 iron–sulphur clusters are consistent with such a mechanism^38^, which is likely to occur during transitions from dark-to-light or low-to-high light transitions. Under these conditions, when PQ is reduced and Fd remains oxidised, electrons from PQ can be routed to O_2_ via Flv proteins, thereby completing a regulatory loop that modulates photooxidation^37^.

In our model, sustained reverse operation of NDH-1 depends on sufficient oxidase flux to provide protons for ATP synthesis while maintaining a proton motive force (pmf) of ~200 mV, which is required to drive the thermodynamically uphill reduction of NADP^+^ via NDH-1^11^. However, maintaining such a high pmf imposes physiological constraints. An excessive transmembrane potential (Δp) can inhibit the Cyt *b*_6_*f* complex, thereby slowing PQH_2_ oxidation and promoting over-reduction of the PQ pool^44^. This redox imbalance may enhance reactive oxygen species production at PSII and reduce the efficiency of proton translocation. In addition, inhibition of Cyt *b*_6_ *f* would limit electron supply to Cox, shifting O_2_ reduction predominantly to Cyd to sustain pmf.

Conversely, an elevated pmf favours reverse NDH-1 activity, which would partially alleviate Cyt *b*_6_ *f* inhibition through pmf dissipation. If reverse NDH-1 activity becomes rate-limiting, an excess of ATP relative to NADPH could constrain ATP synthase turnover, resulting in a transient rise in pmf that further stimulates reverse NDH-1 activity. This feedback interplay suggests a tightly balanced energetic coupling.

Overall, these interdependencies show that PSI‑independent carbon fixation depends on a precisely tuned interplay among terminal oxidases, the Cyt *b*_6_ *f* complex, and ATP synthase, all dynamically coupled through the pmf. Proper coordination of oxidase-driven pmf generation, reverse NDH-1 activity, ATP synthase turnover, and proton dissipation pathways is required to sustain a pmf sufficiently high to support NADPH formation, while avoiding excessive backpressure on Cyt *b*_6_*f* and lumen over-acidification that would compromise electron transport efficiency.

Whether PSI-less photoautotrophy occurs naturally remains an open question. No organisms have yet been identified that combine PSII-dependent water splitting with the absence of a functional PSI. Purple bacteria and filamentous anoxygenic phototrophs also lack PSI, but instead use quinone-type reaction centres in conjunction with cytochrome *bc*_1_ or alternative complex III complexes to generate proton gradients for ATP synthesis. These systems do not extract electrons from water and rely on metabolic redox pathways for reducing power^3^. Thus, the NDH‑1-based PSI bypass described here likely represents a stress-activated alternative electron pathway rather than a naturally occurring photosynthetic state, serving to prevent PQ over-reduction and the associated electron pressure under fluctuating light or metabolic imbalances.

Given that FusA contributes to oxidative stress tolerance and PSII repair^26,27^ and LmbP is annotated as a probable sugar kinase, how might adaptive mutations in *fusA* or *lmbP*, together with changes in at least one additional locus, enable photoautotrophy in the absence of PSI? Two non-exclusive scenarios can be considered. First, this specific set of mutations could modulate the capacity of the proposed NDH-1- and oxidase-dependent electron flow pathways underpinning photoautotrophic growth. Indirect connections, such as translation elongation efficiency constraining respiratory capacity, or LmbP supporting plastoquinone biosynthesis akin to other GHMP kinases, can be envisaged, yet no such mechanisms have been demonstrated, and direct causal links remain elusive. A second explanation is that these mutations primarily mitigate the deleterious effects of PSI loss - by stabilising PSII function, balancing cellular redox state, or improving translational regulation under stress. This interpretation aligns with the higher growth rate, increased PSII abundance and activity, and elevated oxygen evolution observed in *aiao* #1 relative to the Δ*psaAB* control (Figs. 3 and 6), and is consistent with the established roles of FusA in stress tolerance and PSII repair^26,27^. Moreover, adaptive mutations in the elongation factor G homologue FusB/Sll1098 have been shown to enhance high-light tolerance in *Synechocystis* by reducing antenna size^50,51^, and PSI-deficient mutants of *Synechocystis* exhibit decreased photosensitivity when phycobilisomes are removed^33^. Collectively, these findings link adaptive changes in elongation factor G proteins to improved photoprotection in PSI-lacking strains.

Could eukaryotic phototrophs achieve PSI-independent photoautotrophy through an analogous mechanism? In principle, this seems plausible, given the conservation in chloroplasts of key components from the proposed reductant- and proton motive force (pmf)-generating pathways, namely, the NDH-1 complex and oxidases, that underpin photoautotrophic growth in evolved PSI-deficient *Synechocystis* strains. Chloroplast PTOX, however, is non-electrogenic^52^, necessitating introduction of Cyd- or Cox-type oxidases for effective pmf generation. Species-specific variations further challenge direct translation of cyanobacterial adaptations to eukaryotes. In cyanobacteria, adaptive *fusB* mutations mitigate photooxidative stress by limiting phycobilisome accumulation^50,51^, but green algae and higher plants rely on LHC antenna complexes rather than phycobilisomes, rendering such mutations ineffective. Red algae, which retain phycobilisomes^53^, may thus represent more viable candidates. Conversely, PSI deletion in green algae such as *C. reinhardtii* is unlikely to enable photoautotrophy, as these organisms express NDH-2 isoforms lacking proton-pumping activity^6^ and thus insufficient for pmf-driven reverse electron flow.

Decoupling the cellular supply of ATP and NADPH represents a promising strategy to fine-tune energy and redox homoeostasis in a wide range of biotechnological applications. Metabolic pathways with high NADPH demand - whether endogenous processes such as fatty acid biosynthesis and carbon fixation, or heterologous pathways producing highly reduced compounds including biofuels, isoprenoids, and fatty alcohols - could benefit from the enhanced reductant provision mediated by the NDH-1-dependent branch. Likewise, synthetic CO_2_ fixation pathways characterised by elevated NADPH requirements may profit from a partially uncoupled energy and redox supply. Conversely, pmf generation driven by light reactions and terminal oxidases can preferentially support ATP-intensive processes, such as metabolite secretion, protein export, and ion homoeostasis. A controlled allocation of electron flux between redox-generating and ATP-producing modules may additionally limit reactive oxygen species formation, thereby enhancing cellular robustness.

Collectively, these modular and independently adjustable bioenergetic circuits provide a conceptual framework for improving solar-to-chemical energy conversion by dynamically balancing ATP and NADPH fluxes in accordance with specific metabolic demands.

## Methods

### Media and growth conditions

*Synechocystis* strains were cultivated in standard BG11 medium (pH 7.4)^54^ at 30 °C under continuous very low (3 µmol photons m^−2^ s^−1^) or low (30 µmol photons m^−2^ s^−1^) light. Cultures were maintained without shaking or bubbling, as PSI-deficient mutants were sensitive to even mild aeration. When required, BG11 medium was supplemented with 5 mM glucose and appropriate antibiotics.

### Construction of PSI gene-replacement strains

Replacement of native *Synechocystis* PSI subunits with those from *Arabidopsis thaliana* followed published procedures^23,45^. The wild-type genome was modified stepwise through a series of targeted transformations, introducing *A. thaliana* genes and deleting endogenous PSI genes. Marker removal was achieved by counter‑selection or intrachromosomal recombination after each transformation.

Key steps were as follows:

1. Replacement of *psaB* with *AtpsaB* (plasmid pPSI1; selection = kanamycin + sucrose).
2. Replacement of *psaA* with *AtpsaA* (pPSI2; kanamycin + sucrose).
3. Replacement of *psaF/J* with the *AtpsaONHGLIKFJEDC–Specᴿ* cassette (pPSI3; spectinomycin).
4. Sequential deletion of *psaK2*, *psaL/I*, *psaK1* and *psaE* (p∆K2, p∆LI, p∆K1, p∆E; chloramphenicol + sucrose).
5. Deletion of *psaD*,* psaM*, and *psaC* in alternate sequences (p∆D, p∆M, p∆C; chloramphenicol/kanamycin + sucrose) yielding identical final genotypes completely lacking PSI genes (see Fig. 2 and Supplementary Fig. 1).
6. Repair of a transient ~4 kb deletion upstream of *psaF* using plasmid pPSIdel_rev (gentamicin selection). The resulting strain was designated “all‑in, all‑out” (*aiao*).
7. Replacement of *AtpsaA/B* and *AtpsaONHGLIKFJEDC* operons with chloramphenicol‑ and kanamycin‑resistance markers (Cmᴿ, Kanᴿ), producing the PSI‑null strain ΔPSI*.

All gene modifications were verified by PCR and Illumina whole‑genome sequencing (Novogene). To identify the potential mutations of interest, the sequence of the parental strain was used as a baseline from which to subtract background mutations. Only mutations with more than 100x coverage were considered. Large structural variations and deletions were screened using the junction-prediction algorithm in Breseq (v0.39.0)^55^. Deletion mutants of *pgr5* (*ssr2016*) and *ndhB* (*sll0223*) were generated by homologous recombination using pΔsm2^23^ and pΔndhB vectors, respectively. Maps of the DNA constructs used to generate the ΔPSI* and *ndhB* mutant strains are provided in Supplementary Fig. 4.

### Adaptive laboratory evolution (ALE)

To recover photoautotrophic growth in *aiao* backgrounds, four liquid ‘precultures’ (two *aiao* #1, two *aiao* #2) were grown in 150 ml BG11 containing 1.25 mM glucose (25% of standard mixotrophic concentration) at 30 °C with ~10 µmol photons m^−2^ s^−1^. This preliminary cultivation step, carried out prior to the main experiment, served to acclimatize the cultures to the liquid BG11 medium. After 4 days, cells were pelleted (1200 x *g*), divided into triplicates, and resuspended in glucose‑free BG11, resulting in 12 parallel batch cultures. These were maintained under very low fluorescent light (3–5 µmol photons m^−2^ s^−1^) at 30 °C without agitation for 19 weeks. During this period, no additives were provided and the BG11 media was not replenished, with volume loss due to evaporation being minimal due to aluminium foil sealing of the culture flasks. Visible growth appeared after 10–19 weeks in 8 of 12 cultures, which were subsequently sub-cultured and subjected to whole‑genome resequencing.

Δ*psaAB fusA*_*I28T*_ mutants were obtained using a previously established allele swapping protocol^50^, introducing the *fusA*_*I28T*_ point mutation into the endogenous *slr1463* locus of segregated Δ*psaAB* mutants. Maps of the DNA constructs used to generate the Δ*psaAB* and Δ*psaAB fusA*_*I28T*_ mutant strains are provided in Supplementary Fig. 4. For selection of photoautotrophic Δ*psaAB fusA*_*I28T*_ mutants, >100 replicate 50 ml cultures were grown mixotrophically (5 mM glucose) to OD_720nm_ = 0.4–0.6, shifted to low‑glucose (1 mM) medium for 2 days, then transferred to glucose‑free BG11. Cultures were incubated at 3–5 µmol photons m^−2^ s^−1^, 30 °C, and monitored until spontaneous autotrophic growth appeared. One such mutant, Δ*psaAB fusA*_*I28T*_*‑evo*ₐ, was isolated after 15 weeks.

Two additional autotrophic clones, Δ*psaAB fus*_*I28T*_*‑evo*_b_ and Δ*psaAB fus*_*I28T*_*‑evo*_c_, arose under a gradual glucose‑restriction regime. All cultures were grown in 50 ml BG11 medium at 30 °C under continuous illumination of 3 µmol photons m^−2^ s^−1^ without agitation. Starter cultures were supplemented with 1 mM glucose and grown to an optical density at 720 nm (OD_720_) of ~0.3–0.4. Cells were harvested, washed to remove residual glucose, and divided into three independent lineages. When cell density increased markedly (typically after ~10 days for the initial transfer; the interval extended as glucose concentration decreased), cultures were collected again, washed, and resuspended to an OD_720_ of ~0.1. At each subsequent transfer, the glucose concentration was reduced by 10% relative to the preceding step (0.90 mM → 0.81 mM → 0.73 mM, etc.). The process continued until the glucose concentration reached 0.12 mM, at which point no further growth was observed. At each step, aliquots of washed cells were resuspended in glucose‑free BG11 and incubated for at least six months or until growth reappeared. The mutant Δ*psaAB fus*_*I28T*_*‑evo*_b_ exhibited visible growth after 15weeks following transfer from BG11 + 0.90 mM glucose, while mutant Δ*psaAB fus*_*I28T*_*‑evo*_c_ showed growth after 16weeks following transfer from BG11 + 0.73 mM glucose.

### DNA sequencing and mutation analysis

Genomic DNA of evolved, parental, and wild-type strains was isolated using the EasyPure Plant Genomic DNA Kit (TransGen Biotech), sequenced (Illumina, Novogene) and processed using Trimmomatic^56^ for adaptor and quality trimming. Clean reads were aligned to the *Synechocystis* sp. PCC 6803 reference genome (ASM972v1, NCBI) using Bowtie2 (v2.5.1)^57^. Variants were identified with Breseq (v0.39.0)^55^, detecting SNPs, indels, and large rearrangements. Sequencing data have been deposited under NCBI BioProject PRJNA1380123. The complete breseq output files (.gd) along with the indexed alignment files (.bam and.bai) have been uploaded to a Zenodo repository (10.5281/zenodo.18668074).

### Protein immunodetection

Aliquots of 30 ml of cultures (OD_720_ = 1) of cells were pelleted (2400 × *g*, 6 min), resuspended in homogenisation buffer (0.4 M sucrose, 10 mM NaCl, 5 mM MgCl_2_, 20 mM Tricine pH 7.9) containing protease inhibitors, and disrupted with glass beads using a TissueLyser II (Qiagen) across five × 5‑min cycles at 30 Hz. After centrifugation (5000 × *g*, 10 min, 4 °C), supernatants were collected, the protein concentration was determined using the BCA assay (Thermo Fisher Scientific, MA, USA), and the samples analysed by SDS–PAGE (12%). Gels contained ~20 µg total protein per lane. Proteins were transferred to PVDF membranes (0.45 µm, Millipore) and stained with Coomassie Brilliant Blue R‑250. After blocking in 5% milk (TBS‑T), membranes were incubated overnight (4 °C) with primary antibodies against PsaA, PsaB, PsbA, and AtpB. Antibodies (Agrisera, Vännäs, Sweden) were diluted 1:5,000 (anti-PsaA, AS06 172, and anti-PsaB, AS10 695) or 1:10,000 (anti-PsbA, AS05 084 and anti-AtpB, AS05 085).

Detection used SuperSignal West Pico PLUS reagent (Thermo Scientific) and a Fusion FX ECL system (Vilber Lourmat). Immunodetection was performed three times for each antibody using material derived from separate cultures.

### Proteomics

Proteome analysis was performed by the Mass Spectrometry Service Unit (MSBioLMU). Cell lysates were prepared from four independent biological replicates of each strain as described above and solubilized in 6 M guanidine hydrochloride (0.1 M HEPES, pH 8.5) supplemented with protease inhibitors. Samples were incubated at 60 °C for 10 min, sonicated, and clarified by centrifugation at 16,000 × *g* for 15 min. Proteins were digested according to the filter-aided sample preparation protocol^58^. Briefly, 100 µg protein per sample were reduced with 10 mM DTT for 10 min at 37 °C, alkylated with 50 mM iodoacetamide for 20 min in the dark, and digested with sequencing-grade modified trypsin (Promega) at an enzyme-to-substrate ratio of 1:100 overnight at 37 °C. Peptides were recovered and purified using C18 StageTips^59^.

A total of 64 samples were analyzed, comprising 4 biological replicates per condition and 2 technical LC-MS/MS replicates. Samples were analyzed together with quality control and carryover control runs. Blank injections consisting of LC-MS grade water were performed between sample injections to monitor and minimise sample carryover. In addition, bovine serum albumin (BSA) digest quality control samples were analyzed at regular intervals throughout the measurement sequence to monitor chromatographic performance, mass accuracy, sensitivity, and instrument stability.

For LC-MS/MS analysis, 1 µg peptide was loaded onto an Ultimate 3000 RSLC nanoLC system (Thermo Fisher Scientific). Peptides were separated on a reversed-phase C18 analytical column (75 µm × 50 cm, 100 Å pore size, 5 µm particle size) maintained at 50 °C. Solvent A consisted of water containing 0.1% formic acid and solvent B consisted of acetonitrile containing 0.1% formic acid. Peptides were eluted using a linear gradient from 5% to 80% solvent B over 40 min at a flow rate of 250 nL min⁻¹.

The LC system was coupled online to an Impact II quadrupole time-of-flight mass spectrometer (Bruker Daltonics) equipped with a CaptiveSpray ion source and operated in positive ion mode. Data were acquired in data-dependent acquisition mode. Full MS scans were recorded from m/z 200–2000 at a scan rate of 3 Hz. The 18 most intense precursor ions exceeding an intensity threshold of 500 counts were selected for CID fragmentation and acquired at a scan rate of 20 Hz. Singly charged ions were excluded from precursor selection and ions with charge states 2+ to 6+ were preferentially selected. Dynamic exclusion was enabled with an exclusion duration of 30 s and precursor re-inclusion factor of 3. Fragmentation was performed using collision-induced dissociation with instrument-controlled collision energies.

Raw data were processed using MaxQuant v2.4.14.0 with the integrated Andromeda search engine. Spectra were searched against a custom protein sequence database comprising *Synechocystis* proteins and *Arabidopsis* PSI subunits, supplemented with the MaxQuant contaminant database. Enzyme specificity was set to Trypsin/P allowing up to two missed cleavages. Carbamidomethylation of cysteine residues was specified as a fixed modification, while oxidation of methionine and protein N-terminal acetylation were included as variable modifications. A maximum of five modifications per peptide was allowed. The minimum peptide length was set to seven amino acids and the maximum peptide mass to 4,600 Da. Precursor mass tolerances were set to 20 ppm for the first search and 10 ppm for the main search. Peptide-spectrum matches, peptides, proteins and modification sites were filtered using a target-decoy approach to a false discovery rate of 1%. Modified peptides were required to have a minimum Andromeda score of 40 and a minimum delta score of 6. Protein groups were reported with a minimum of one peptide, including razor peptides. Label-free quantification was performed using the MaxLFQ algorithm with a minimum ratio count of 2. Match-between-runs and dependent peptide searches were disabled^60^.

Label-free protein quantification was performed using the MaxLFQ algorithm implemented in MaxQuant with a minimum ratio count of 2 and otherwise default settings. Proteins identified as contaminants, reverse hits, or only identified by site were excluded from downstream analyses.

The mass spectrometry proteomics data have been deposited to the ProteomeXchange Consortium via the PRIDE partner repository under accession number PXD071953.

### Pigment quantification

Chlorophyll *a* and carotenoids were extracted from cultures (OD_720nm_ = 0.75; using three biological replicates from each of three independently grown cultures per strain, nine replicates in total per strain) using 100% methanol and quantified spectrophotometrically at 470, 665, and 720 nm^61^. Phycocyanin was isolated after cell disruption in PBS (pH 7.4) with glass beads using a TissueLyserII (Qiagen) at 30 Hz for 5 min. The lysate was then centrifuged twice at 4 °C, first at 4000 × *g* for 5 min, and subsequently at 16000 × *g* for 5 min using the collected supernatant. The absorbance of the resulting supernatant was measured at 615 and 652 nm^62^, and pigment concentrations were determined using standard calculation formulas.

### Dry mass measurements

Ten ml of culture were pelleted, dried overnight at 65 °C, and then weighed using an analytical balance (KERN, Germany). Mixotrophic and autotrophic cultures (three independent samples for each strain) were harvested after 7 and 14 days, respectively.

### Analysis of ^13^C‑labelled sucrose

Cultures were grown to an OD_720nm_ of 0.4–0.6 in BG11 containing 5 mM glucose. 100 ml of each culture (three biological replicates for each strain) were collected, the glucose washed out, and the cells were resuspended in fresh BG11 (120 ml, 250 ml bottle). NaH^13^CO_3_ (15 mg) or NaHCO_3_ (control) was added, and cultures were incubated 72 h at 30 °C, 3 µmol photons m^−2^ s^−1^.

Sucrose analysis was performed with modifications based on previous protocols^63–66^. Briefly, pelleted cells were thoroughly mixed with 180 µl of cold methanol ( − 20 °C) in a 1.5 ml microcentrifuge tube containing internal standards: 5 μl of ribitol (0.2 mg ml⁻¹ in water) and 5 μl of ¹³C-sorbitol (0.2 mg ml⁻¹ in water). The mixture was incubated at 70 °C for 15 min with continuous mixing (950 rpm), then cooled to room temperature. Next, 100 μl of chloroform followed by continuous mixing (950 rpm) and 200 µl of water were added to induce phase separation, followed by strong mixing and centrifugation at maximum speed for 15 minutes.

The upper (polar) phase (350 µl) was collected and dried under vacuum in a 1.5 ml tube. The resulting residue was resuspended in 100 µl of methanol:water (50:50 v/v), transferred to a 200 µl glass vial and dried again under vacuum. The dried extracts were stored at −20 °C until derivatization.

For derivatization, the dried residue was resuspended in 10 μl of methoxyamine hydrochloride (20 mg ml⁻¹ in pyridine) and incubated at 40 °C for 90 min. Subsequently, 20 µl of BSTFA (N,O-Bis[trimethylsilyl]trifluoroacetamide) containing 2.5 μl of a retention index standard mix of linear alkanes (n-decane, n-dodecane, n-pentadecane, n-nonadecane, n-docosane, n-octacosane, and n-dotriacontane) was added. The mixture was further incubated at 40 °C for 45 minutes.

One microliter of the derivatized sample was injected into a GC–TOF–MS system (Pegasus HT, Leco, St. Joseph, USA) via an autosampler (Combi PAL, CTC Analytics AG, Zwingen, Switzerland). Helium was used as the carrier gas at a constant flow rate of 1 ml min⁻¹. Gas chromatography was performed using an Agilent 7890 A GC system equipped with a 30 m VF-5ms capillary column and a 10 m EZ-Guard pre-column. The injector, transfer line, and ion source temperatures were all maintained at 250 °C. The oven temperature was initially set at 70 °C and ramped to 350 °C at 9 °C min⁻¹.

Metabolites were ionised using electron impact at 70 eV and detected at a scanning rate of 20 spectra per second across a mass range of m/z 50–600. Data acquisition and processing were carried out using ChromaTOF software version 4.72.

For quantification, we used the sucrose fragment with a monoisotopic mass of *m/z* 361, which contains six possible isotopologues (*m/z* 362–367), each carrying one additional heavy isotope.

Upon incorporation of ^13^CO_2_, the relative abundance of the heavier isotopologue peaks (m/z 362–367) increases compared to the monoisotopic peak (m/z 361). Labelling was performed using ^13^C-bicarbonate, while ¹²C-bicarbonate served as the control. Photosynthetically active strains display a clear shift of the MID toward the heavier isotopologues during ^13^C-bicarbonate feeding, whereas photosynthetically inactive strains retain the MID characteristic of the ^12^C controls.

All measured peak intensities were normalised to the total sucrose signal (sum of *m/z* 361–367). The monoisotopic and isotopologue fractions therefore represent their relative percentages within the total MID.

### Low‑temperature fluorescence

77 K fluorescence spectra were recorded using a HORIBA Fluoromax Plus spectrofluorometer. Cell suspensions (OD_720nm_ = 2.0) were frozen in glass capillaries by immersion in liquid N_2_. Excitation wavelengths of 438 nm (Chl *a*) and 590 nm (phycobilisomes) were used, with signal integration at 0.2 s nm^−1^. Spectra were normalised to the 600 nm fluorescence peak. Each measurement was performed on individually grown cultures, with *n* = 3 for *aiao-evo*_ext_, *n* = 7 for Δ*psaAB* and *n* = 4 for the remaining strains.

### Oxygen evolution

Oxygen production was measured using a Clark‑type Oxytherm+ P electrode (Hansatech Instruments). For net oxygen evolution measurement, cultures (OD_720nm_ ≈ 0.6) were adjusted to OD_720nm_ = 1.2 in fresh BG11 in a final sample volume of 2.0 ml and dark‑adapted before measurement. Oxygen concentration was recorded every 0.1 s during 5 min illumination ( ≈ 80 µmol photons m^−2^ s^−1^, 2% instrument power), followed by 5 min dark incubation. Four independent cultures were measured for each strain. To measure PSII activity specifically, the inhibitors DCBQ (2,6-Dichloro-p-benzoquinone) and K_3_Fe(CN)_6_ were added to the final concentrations of 0.5 mM and 1 mM, respectively. Cultures were resuspended to OD_720nm_ = 2 to a final sample volume of 1.5 ml and illuminated for 4 min ( ≈ 80 µmol photons m^−2^ s^−1^, 2% instrument power), followed by 1 min of dark incubation.

### P515 measurements

P515 changes were monitored using a DUAL-PAM100 fluorospectrometer (Walz, Germany) with a P515/535 module as described^36^. Cultures were grown to OD_720nm_ of 0.4-0.6 (in BG11 + 5 mM glucose, 30 °C, 3 µmol photons m^−2^ s^−1^), then washed and adjusted to an OD_720nm_ of 2. The cultures were then aliquoted into 2 ml tightly closed tubes. Cultures were dark-incubated for at least 16 h prior to measurement. The following DUAL-PAM protocol was used: 15 s dark, 600 s red actinic light (216 μmol m^−2^ s^−1^; 635 nm), 135 s dark. The measuring light intensity was 5, and the data acquisition rate was 1,024,000 per 20 ms. For each strain, the measurement was repeated four times, using the separate samples.

### NADP^+^ reduction measurements

NADPH levels were recorded using a DUAL-PAM100 fluorospectrometer (Walz, Germany) equipped with a NADPH/9-AA module. Cultures were grown to an OD_720nm_ of 0.4-0.6 (BG11 + 5 mM glucose; 30 °C, 3 µmol photons m^−2^ s^-^^1^), harvested, washed, and resuspended in iron-depleted BG11 medium to a final OD_720nm_ of 2. Prior to measurements, cultures were dark-adapted for at least 16 h. The following DUAL-PAM protocol was used: 20 s dark, four cycles of 120 s light-60 s dark, finished with 120 s light and 20 s dark. The settings were as follows: red actinic light intensity 1 (6 μmol m^2^ s^−1^; 635 nm); gain 1 (Low); damping 10 μs (Low); Block Frequency MF-low 200 and MF-high 5000; NADPH-Fluo Meas. Light 20; the amplifier was set to the values 4 (coarse) and 8 (fine).

### Calculations of proton stoichiometry

All calculations were normalised to a basis of two transferred electrons (2 e⁻). Proton translocation stoichiometries were assigned as follows: +2 H^+^ from luminal release from PSII water oxidation^67^, +4 H⁺ from cytochrome *b*_6_*f* complex via the Q-cycle^68,69^, and −4 H⁺ from reverse electron flow through NDH-1^11,70^. For respiratory pathways the net proton yield per 2 e⁻ (including PSII and, where applicable, Cyt *b*_6_*f*) was defined as +4 H⁺ for the Cyd oxidase pathway^71^ and +10 H⁺ for the Cox oxidase pathway^72^. A mixed scenario assuming concurrent and equal flow through Cyd and Cox resulted in an average net translocation of +7 H⁺ per 2 e⁻.

### Modelling electron partitioning for pmf maintenance and metabolic demand balancing

To determine the electron flux required to sustain pmf, we defined $x$ as the fraction of total electron flux directed to reverse NDH-1, with the remaining $(1-x)$ allocated to oxidase pathways. The threshold for pmf maintenance $(\Delta {H}^{+} > 0)$ was derived using the following regime-specific linear equations:

Mixed (Cyd + Cox): $\Delta {H}^{+}=7(1-x)-2x=7-9x.$ $\Delta {H}^{+}=7-9x > 0$ gives $x < 0.778$ implying that ≥ 22.2 % of electron flux must pass through the oxidases to prevent pmf dissipation.

With:

$${{\rm{Cyd}}}{\mbox{-}}{{\rm{only}}}:\Delta {H}^{+}=4-6x$$

$${{\rm{Cox}}}{\mbox{-}}{{\rm{only}}}:\Delta {H}^{+}=10-12x$$

corresponding calculations result in $x < 0.667$ ( ≥ 33.3% oxidase flux) for Cyd, and $x < 0.833$ ( ≥ 16.7% oxidase flux) for Cox.

To model metabolic demand balancing, ATP synthesis was modelled assuming a c-ring stoichiometry of 14 H⁺ per 3 ATP, equivalent to 4.67 H⁺ per ATP^73^. The ATP yield per 2 e⁻ was thus computed as: ATP = ΔH^+^/4.67. To meet the stoichiometric demand of the Calvin–Benson cycle, the ATP/NADPH ratio was fixed at 3:2 (1.5)^74^. Electron partitioning (*x*) was solved algebraically by setting the ratio of ATP yield to NADPH generation (*x*) equal to this demand.

$$\frac{{\mbox{ATP}}}{{\mbox{NADPH}}}=\frac{\Delta {H}^{+}}{4.67x}=1.5$$

For the mixed oxidase regime, the net proton translocation per 2 e⁻ is:

$$\Delta {H}^{+}=7-9x$$

giving an overall ratio:

$$\frac{{\mbox{ATP}}}{{\mbox{NADPH}}}=\frac{7-9x}{4.67x}.$$

Setting this to 1.5 yields $x=0.437$, which corresponds to a net proton translocation of 3.07 H⁺ per 2 e⁻ and an ATP yield of 0.66 per 2 e⁻. With respect to oxygen exchange, extraction of two electrons from water at PSII results in the evolution of 0.5 molecules of O_2_. The fraction of electrons not directed toward NADP^+^ reduction, (1 − *x*), is instead transferred to terminal oxidases, where reduction of 0.5 O_2_ requires 2 electrons. Accordingly, the oxidase branch consumes 0.5(1 − *x*) molecules of O_2_ per 2 e⁻ withdrawn from water. Net oxygen production is therefore given by 0.5 − 0.5(1 − *x*) = 0.5*x*.

Steady-state solutions for *x*, the net change in H⁺, ATP yield and net O_2_ production per 2 e⁻ involving either Cyd or Cox oxidase activity alone were calculated correspondingly, employing the corresponding equations for ΔH⁺.

### Statistical analysis

Proteomic data were analysed in Perseus v2.1.5^75^. Proteins with ≥ 3 valid intensity values were retained, log_2_‑transformed, and missing values imputed (1.8 SDshift, 0.3 SDwidth). Significance was determined by ANOVA with FDR correction (q < 0.05, |log_2_ fold change | >0.58); pairwise differences were evaluated with Tukey’s HSD test. For pigment and dry‑mass comparisons, one‑way ANOVA (two‑sided) with Holm‑corrected Tukey HSD was applied. For isotopologue distributions, ANOVA followed by Duncan’s multiple range test was used; detailed statistics are provided in Source Data.

### Accession numbers

PSI subunits of *Arabidopsis thaliana* and *Synechocystis* sp. PCC 6803 are listed. “-” indicates the absence of a corresponding homologue in plants or cyanobacteria. PsaA, AtCg00350/Slr1834; PsaB, AtCg00340/Slr1835; PsaC, ArthCp075/Ssl0563; PsaD1, At4g02770/Slr0737; PsaE1, At4g28750/Ssr2831; PsaF, At1g31330/Sll0819; PsaG, At1g55670/-; PsaH1, At3g16140/-; PsaI, ArthCp032/Smr0004; PsaJ, AthCp042/Sml0008; PsaK, At1g30380/Ssr0390_sll0629; PsaL, At4g12800/Slr1655; PsaM, -/Smr0005; PsaN At1g49975/-; PsaO At1g08380/-. Additional *Synechocystis* genes:* fusA* (Slr1463), *ndhB* (Sll0223), *pgr5* (Ssr2016).

### Reporting summary

Further information on research design is available in the Nature Portfolio Reporting Summary linked to this article.

## Supplementary information

Supplementary information

Reporting Summary

Transparent Peer Review file

## Source data

Source Data

## Data Availability

Raw genome re-sequencing data is available on the NCBI SRA, Project no. PRJNA1380123; [https://dataview.ncbi.nlm.nih.gov/object/PRJNA1380123?reviewer=372a9hs11f9f4521c95mo5qr6m]. breseq output files can be accessed on Zenodo [10.5281/zenodo.18668074]. Proteomics data are available via PRIDE PXD071953. Source data are provided with this paper.
