# Supplementary material for: Photosystem I-independent oxygenic photosynthesis in cyanobacteria
Source: Nat Commun. 2026 Jul 10;17:6081. doi: 10.1038/s41467-026-74903-2 (PMC13354564; doi:10.1038/s41467-026-74903-2)
Supplement: Supplementary file 1 — Supplementary information [file 41467_2026_74903_MOESM1_ESM.pdf]

SUPPLEMENTARY

Photosystem I-independent oxygenic photosynthesis in cyanobacteria

Marta Ludwiczak, Marcel Dann, Theo Figueroa-Gonzalez, Eslam M. Abdel-Salam, Weiyang Chen, Serena Schwenkert, Martin Lehmann, Milena Zhivkovikj, Maysoon Noureddine, Markéta Linhartová, Sadanand Gupta, Josef Komenda, Arthur Guljamov, Stefania Viola, Feng Liu & Dario Leister

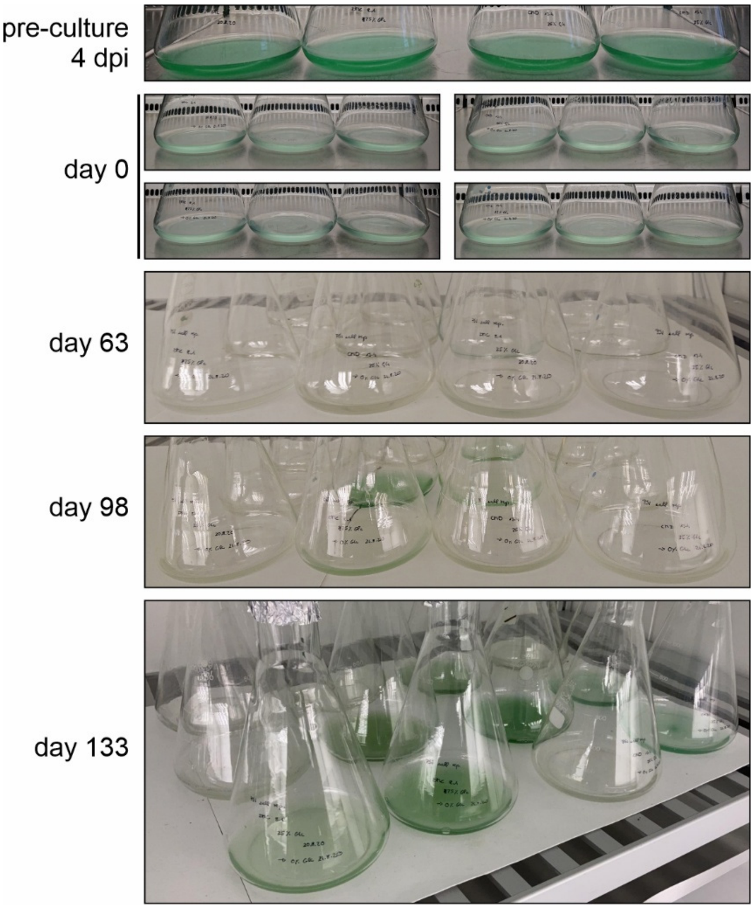

Supplementary Fig. 1 │ Time course of the 19-week adaptive laboratory evolution (ALE) experiment yielding *aiao-evo* strains.

Two replicate cultures of *aiao* #1 and *aiao* #2 were pre-cultured in antibiotic-free BG11 medium containing 1.25 mM glucose (25% of the standard concentration; top panel). Four days post-inoculation, cells were harvested and divided into three identical aliquots, each diluted back to the original volume with glucose-free BG11 and transferred to low-light conditions (~3 µmol µmol photons m^{-2} s^{-1}). Most cells died within ~10 days, leaving the medium transparent, and no visible growth occurred for ≈ 63 days. Photoautotrophic growth first became evident after ≈ 98 days. After ≈ 133 days, eight of twelve culture flasks exhibited varying levels of green biomass accumulation.

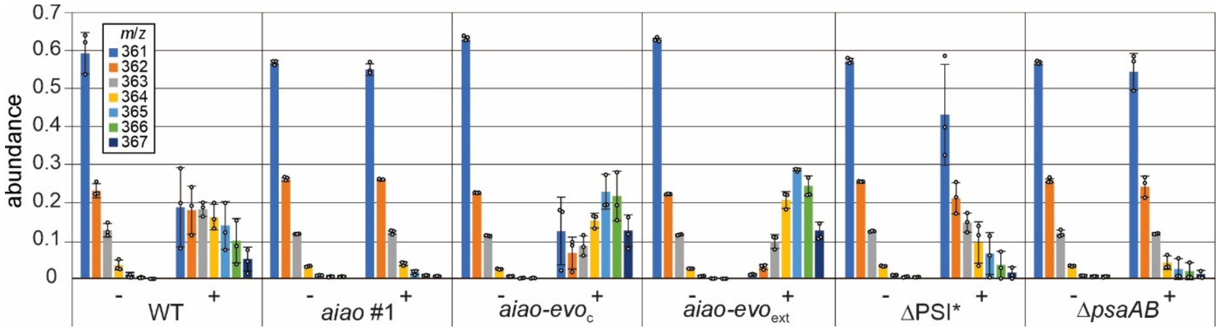

Supplementary Fig. 2 │ Sucrose isotopomer distributions following bicarbonate feeding cells with either uniformly ^{13}C (+)- or natural abundance (-) isotope composition (see Fig. 6a). The relative abundance of isotopomers (M+0 to M+6, *m*/*z* 361-367) is shown for each genotype, left to right. *N* = 3 for each strain; all calculations and statistics are provided in the Source data file.

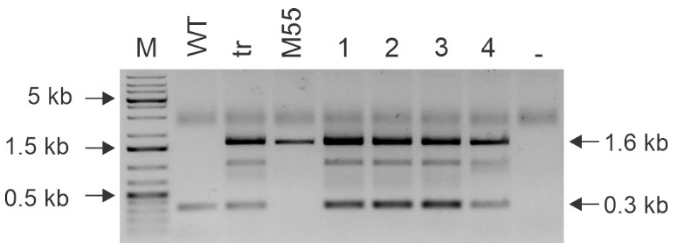

Supplementary Fig. 3 │ PCR-based genotyping of the *ndhB* locus (see Fig. 7).

In the *aiao-evo*c strain, the *ndhB* gene was disrupted by a kanamycin resistance cassette. Transformants were initially selected on BG11 medium supplemented with 5 mM glucose and 10 µg mL^{-1} kanamycin, followed by stepwise re-streaking onto plates containing 10–300 µg mL^{-1} kanamycin. "WT" indicates the wild-type strain; "M55" corresponds to the WT Δ*ndhB* mutant; and "tr" designates un-segregated transformants. Colonies 1–4 were obtained from transformants maintained on 5 mM glucose and 300 µg mL^{-1} kanamycin. For genotyping, cells were directly lysed in PHIre Plant Direct PCR buffer (Thermo Fisher Scientific, MA, USA), and amplified per the manufacturer's instructions. PCR products of 0.3 kb (primers M55check_FW: GCCAGAGGGCATTGTTATT; M55check_RV: AGGTAGCCGTGAGCAAAATG) indicate presence of the wild-type *ndhB* copy, whereas a 1.6 kb band denotes integration of the kanamycin cassette.

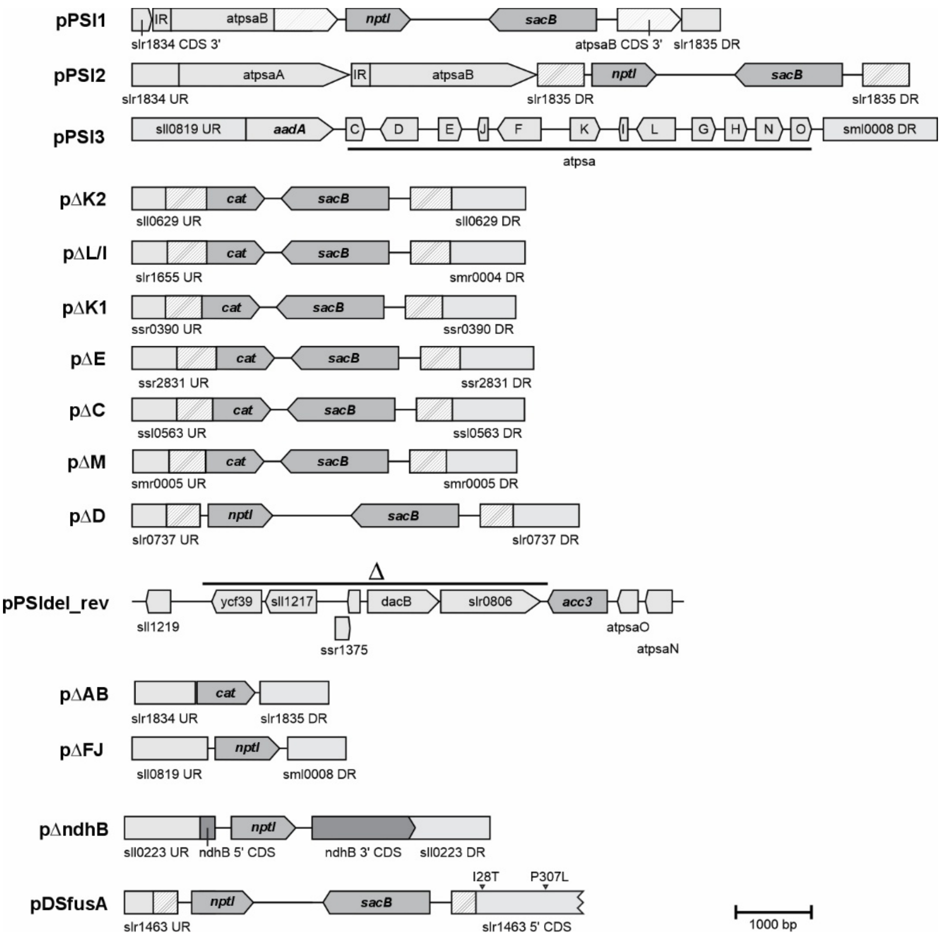

Supplementary Fig. 4 │ Genetic constructs used for generation of *Synechocystis* PSI mutant strains.

Schematic maps are drawn to scale (base pairs, see scale bar). Regions of internal homology used for intrachromosomal recombination are shown with diagonal shading. Selection markers: *nptI*, kanamycin; *aadA*, spectinomycin; *cat*, chloramphenicol; *acc3*, gentamycin. The counter-selectable marker *sacB* confers sucrose sensitivity. Synthetic *Arabidopsis* PSI small-subunit gene cluster (pPSI3) and restored chromosomal deletion fragment (pPSIdel_rev) are shown as bold horizontal lines. UR, upstream region; DR, downstream region; CDS, coding sequence. Construct nucleotide sequences are provided in the source data file (**Source Data**).

Supplementary Table 1 | Average sequencing coverage (×) per replicon for each strain.
Mean read depth across the chromosome and plasmids is shown.

**Strain**	**Chromosome**	**pSYSA**	**pSYSG**	**pSYSM**	**pSYSX**
WT	310.4	72.4	130.7	170.5	231.5
aiao #1	169.0	82.7	106.3	215.7	190.0
aiao-evo$_a$	165.9	47.7	37.4	182.3	175.5
aiao-evo$_b$	145.2	66.3	82.7	192.3	179.7
aiao-evo$_c$	116.1	39.4	49.4	138.4	129.2
aiao-evo$_d$	152.8	46.9	61.0	158.1	144.3
aiao #2	124.1	52.0	71.4	140.1	118.1
aiao-evo$_e$	143.7	36.5	61.8	146.8	126.5
aiao-evo$_f$	144.2	42.4	62.4	149.8	138.6
aiao-evo$_g$	159.6	51.7	79.2	181.3	164.5
aiao-evo$_h$	202.2	45.0	58.9	215.9	205.8

Supplementary Table 2 │ Read mapping efficiency relative to the reference genome. For each strain, the proportion (%) of total sequencing reads aligning to the *Synechocystis* PCC 6803 genome (ASM972v1) is reported.

**Strain**	**Mapped reads (%)**
WT	98.73
aiao #1	97.96
aiao-evo$_{a}$	99.18
aiao-evo$_{b}$	99.39
aiao-evo$_{c}$	99.49
aiao-evo$_{d}$	99.44
aiao #2	98.37
aiao-evo$_{e}$	99.07
aiao-evo$_{f}$	99.35
aiao-evo$_{g}$	99.29
aiao-evo$_{h}$	99.49

Supplementary Table 3 | *Pre-evolution* mutations in *aiao* strains.

Mutations present in both *aiao* and *aiao-evo* cultures, but absent from the WT, are listed. Each affected gene contains at least one mutant allele with an allele frequency exceeding >20%. These represent *pre-evolution* variants that arose during the construction and prolonged maintenance of the *aiao* strains, prior to the subsequent evolution of photoautotrophy in the *aiao-evo* lineages.

Gene/locus	Annotation	Strain	Codon mutation	Amino acid change	Frequency of mutation [%]
ilvB (*slr2088*)	acetolactate synthase subunit	*aiao* #1	ATG→GTG	M280V	100
		aiao #2			100
rsmG (*slr0072*)	16S rRNA (guanine(527)-N(7))-methyltransferase	*aiao* #1	+A (706/795 nt)	frameshift	88.25
		aiao #2	TGG→TAG	W35stop	100
sll0296	Uma2 family endonuclease	*aiao* #2	CAT→TAT	H41Y	48.88
intergenic region *slr0397* → / → *slr0398*		*aiao* #1	C→A	intergenic (+17/-155)	100
		aiao #2			100
slr0806	FAD-binding oxidoreductase	*aiao* #2	GAA→AAA	E123K	54.67
slr6090	cyanobacterial Rep-related protein	*aiao* #1	AGG→AAG	R885K	9.89
		aiao #2			12.39
		aiao #1	AAG→AAA	K894K	12.35
		aiao #2			20.02
		aiao #1	GAA→AAA	E1016K	28.25
		aiao #2			32.34
		aiao #1	GGC→AGC	G1012S	28.29
		aiao #2			30.04
		aiao #1	CAC→CAT	H1009H	32.38
		aiao #2			36.02
		aiao #1	GCA→GAA	A919E	32.96
		aiao #2			34.23
		aiao #1	ACC→GCC	T1007A	36.89
		aiao #2			35.79
		aiao #1	ATC→GTC	I999V	42.52
		aiao #2			41.18
