# Supplementary material for: Photosystem I-independent oxygenic photosynthesis in cyanobacteria
Source: Nat Commun. 2026 Jul 10;17:6081. doi: 10.1038/s41467-026-74903-2 (PMC13354564; doi:10.1038/s41467-026-74903-2)
Supplement: Supplementary file 2 — Reporting Summary [file 41467_2026_74903_MOESM2_ESM.pdf]

nature portfolio

Corresponding author(s): Dario Leister

Last updated by author(s): Jun 11, 2026

Reporting Summary

Nature Portfolio wishes to improve the reproducibility of the work that we publish. This form provides structure for consistency and transparency in reporting. For further information on Nature Portfolio policies, see our Editorial Policies and the Editorial Policy Checklist.

Statistics

For all statistical analyses, confirm that the following items are present in the figure legend, table legend, main text, or Methods section.

n/a	Confirmed	
☐	☒	The exact sample size (*n*) for each experimental group/condition, given as a discrete number and unit of measurement
☐	☒	A statement on whether measurements were taken from distinct samples or whether the same sample was measured repeatedly
☐	☒	The statistical test(s) used AND whether they are one- or two-sided<br>*Only common tests should be described solely by name; describe more complex techniques in the Methods section.*
☒	☐	A description of all covariates tested
☐	☒	A description of any assumptions or corrections, such as tests of normality and adjustment for multiple comparisons
☐	☒	A full description of the statistical parameters including central tendency (e.g. means) or other basic estimates (e.g. regression coefficient) AND variation (e.g. standard deviation) or associated estimates of uncertainty (e.g. confidence intervals)
☐	☒	For null hypothesis testing, the test statistic (e.g. *F*, *t*, *r*) with confidence intervals, effect sizes, degrees of freedom and *P* value noted<br>*Give P values as exact values whenever suitable.*
☒	☐	For Bayesian analysis, information on the choice of priors and Markov chain Monte Carlo settings
☒	☐	For hierarchical and complex designs, identification of the appropriate level for tests and full reporting of outcomes
☒	☐	Estimates of effect sizes (e.g. Cohen's *d*, Pearson's *r*), indicating how they were calculated

Our web collection on statistics for biologists contains articles on many of the points above.

Software and code

Policy information about availability of computer code

Data collection	77K fluorescence spectra were collected using FluorEssence™ Software (HORIBA Scientific, Oberursel, Germany)<br>Enhanced chemiluminescence data was collected using FUSION FX software (Vilber, Collégien, France)<br>Clark-type oxygen electrode current data was collected using OxyTrace+ software (Hansatech, Pentney, UK)<br>Proteomics mass spectra were collected using an Impact II Q-TOF mass spectrometer (Bruker Daltonics, Billerica, USA) equipped with a CaptiveSpray ESI source, coupled to an Ultimate 3000 RSLC nano-LC system (Thermo Fisher Scientific, Waltham, USA).<br><br>Metabolite mass spectra were collected using a Pegasus HT GC–TOF–MS system (Leco, St. Joseph, USA) coupled to an Agilent 7890A gas chromatograph (Agilent Technologies, Santa Clara, USA) and an autosampler (Combi PAL, CTC Analytics AG, Zwingen, Switzerland).<br>NADPH fluorescence and P515 absorbtion kinetics data were collected with a DUAL-PAM-100 (Heinz Walz GmbH, Effeltrich, Germany) equipped with the NADPH fluorescence module and the P515/535 emitter–detector module using the PC software DualPAM V3.32. Raw data were exported as CSV files using the native DualPAM software interface.
Data analysis	the quality of the WGS raw data was assessed using FastQC v0.11.9<br>pre-processing was performed using Cutadapt v4.1<br>k-mer coorection was performed using Rcorrector<br>mutation detection was performed using the Breseq pipeline<br>clean reads were aligned to the Synechocystis sp. PCC 6803 reference genome (ASM972v1) using bowtie2 v2.5.1<br>polymorphic and fixed mutations were identified by Breseq<br>77K spectra O2 data and NADPH fluorescence/P515 absorption kinetics were analysed in Microsof Excel

Proteomics data analysis was performed using MaxQuant v2.4.14.0 with default parameters and label-free quantification (LFQ) via the MaxLFQ algorithm against the Synechocystis proteome (UniProt UP000889800) supplemented with Arabidopsis PSI proteins.

Metabolomics data analysis was performed using ChromaTOF software (v4.72) for data acquisition and processing, and TagFinder (v4.1) for metabolite identification and ^{13}C-enrichment analysis.

For manuscripts utilizing custom algorithms or software that are central to the research but not yet described in published literature, software must be made available to editors and reviewers. We strongly encourage code deposition in a community repository (e.g. GitHub). See the Nature Portfolio guidelines for submitting code & software for further information.

Data

Policy information about availability of data

All manuscripts must include a data availability statement. This statement should provide the following information, where applicable:
- Accession codes, unique identifiers, or web links for publicly available datasets
- A description of any restrictions on data availability
- For clinical datasets or third party data, please ensure that the statement adheres to our policy

All source data of this study is provided in the supplementary data sets. Biological material is available upon request.

DNA sequence data: Reviewer access: https://dataview.ncbi.nlm.nih.gov/object/PRJNA1380123?reviewer=372a9hs11f9f4521c95mo5qr6m

Reviewer access details for proteomics data: Log in to the PRIDE website using the following details:
Project accession: PXD071953
Token: FFyihTq71TTd
Alternatively, reviewer can access the dataset by logging in to the PRIDE website using the following account details:
Username: reviewer_pxd071953@ebi.ac.uk
Password: C49RjSN5WO2L

Research involving human participants, their data, or biological material

Policy information about studies with human participants or human data. See also policy information about sex, gender (identity/presentation), and sexual orientation and race, ethnicity and racism.

Reporting on sex and gender	not applicable
Reporting on race, ethnicity, or other socially relevant groupings	not applicable
Population characteristics	not applicable
Recruitment	not applicable
Ethics oversight	not applicable

Note that full information on the approval of the study protocol must also be provided in the manuscript.

Field-specific reporting

Please select the one below that is the best fit for your research. If you are not sure, read the appropriate sections before making your selection.

☒ Life sciences ☐ Behavioural & social sciences ☐ Ecological, evolutionary & environmental sciences

For a reference copy of the document with all sections, see nature.com/documents/nr-reporting-summary-flat.pdf

Life sciences study design

All studies must disclose on these points even when the disclosure is negative.

Sample size	wherever possible, at least three biological replicates were assayed.
Data exclusions	no data was excluded from the analyses
Replication	Experimental findings were replicated at least trice (n>=3). All data presented could be reproduced within the range determined through given average values and stadard deviations.
Randomization	not applicable
Blinding	not applicable

Reporting for specific materials, systems and methods

We require information from authors about some types of materials, experimental systems and methods used in many studies. Here, indicate whether each material, system or method listed is relevant to your study. If you are not sure if a list item applies to your research, read the appropriate section before selecting a response.

Materials & experimental systems

n/a	Involved in the study	
☐	☒	Antibodies
☒	☐	Eukaryotic cell lines
☒	☐	Palaeontology and archaeology
☒	☐	Animals and other organisms
☒	☐	Clinical data
☒	☐	Dual use research of concern
☒	☐	Plants

Methods

n/a	Involved in the study	
☒	☐	ChIP-seq
☒	☐	Flow cytometry
☒	☐	MRI-based neuroimaging

Antibodies

Antibodies used	antibodies used in this study were obtained from Agrisera (anti-AtpsaA: AS06 172; anit-AtpsaB: AS10 695; anti-AtpsbA: AS05 084; Anti-AtAtpB: AS05 085; anti.NdhB: AS16 4064) (Agrisera, Vännäs, Sweden)
Validation	Agrisera antibodies as per their respective datasheets

Plants

Seed stocks	not applicable
Novel plant genotypes	not applicable
Authentication	not applicable
