# Supplementary material for: Photosystem I-independent oxygenic photosynthesis in cyanobacteria
Source: Nat Commun. 2026 Jul 10;17:6081. doi: 10.1038/s41467-026-74903-2 (PMC13354564; doi:10.1038/s41467-026-74903-2)
Supplement: Supplementary file 3 — Transparent Peer Review file [file 41467_2026_74903_MOESM3_ESM.pdf]

nature portfolio

Peer Review File

Photosystem I-independent oxygenic photosynthesis in cyanobacteria

Corresponding Author: Professor Dario Leister

This file contains all reviewer reports in order by version, followed by all author rebuttals in order by version.

Version 0:

Reviewer comments:

Reviewer #1

(Remarks to the Author)
Ludwiczak et al. report the serendipitous discovery of strains of Synechocystis sp. PCC 6803 capable of photosynthetic growth in the absence of photosystem I. The authors provide a full characterisation of various engineered and evolved strains using genomics and photosynthesis experiments, showing that there is a degree of photosynthetic growth in some of these strains in the absence of PSI. Restoration of photosynthetic growth is attributed to the emergence of mutations on the translation elongation factor G (FusA) and another uncharacterised protein. It is suggested that electrons flow in reverse via NDH-1 to ferredoxin to sustain carbon fixation.

I find the data presented sufficiently compelling to support the conclusion of restoration of photosynthetic growth in the absence of PSI. The role of NDH-1, while a likely explanation and a valid hypothesis, I think it is only indirectly supported with the presented data, which I find adequate at this stage.

I do not have any major concerns. Below I provide some comments aimed at improving clarity:

Lines 85-89. What is your best hypothesis for why the aiao#1 and aiao#2 strains are strictly heterotrophic despite containing a full complement of PSI genes? What about your engineering approach aimed at introducing PSI from A. thaliana means that there is no expression despite successful integration? I think it is appropriate to address this issue in the discussion.

Line 89. I think some of the supplementary data/table/figures in the provided excel file requires explanatory legends. Please add to all cases in which this is missing. Do not assume that anything is self-explanatory.

Line 123. “putative sugar kinase,” I’m not sure this is accurate enough. Please be more accurate on how homologues of this gene are annotated beyond Synechocystis 6803.

Line 176. It is implied that the deletion of the ndhB gene in the wild-type strain does not abolish photosynthesis. Is this the case? Please state or discuss with appropriate citations.

Discussion
Lines 191-195. Adding more results at the start of the discussion is confusing. There is also ambiguity and vagueness on what you report here. Please rephrase/restructure as necessary.

Line 196. I think you should be more cautious about how you present the data and interpretations on the role of NDH-1. One could argue that your strains are already at the edge of survivability under photosynthetic conditions, and I think it is possible that any further perturbation could tip them over. Unless you measure electron transfer from PQ to Fd via NDH-1, I think you should keep this attribution as a hypothesis and toned down.

Line 217. “this unique combination of adaptive mutations…” What combination of mutations do you mean exactly?

Line 219, 220. “the mutations…” be more specific, what mutations exactly?

Out of the two explanations you suggest, the first one does not make much sense. At least you do not provide a rationale for how this could be possible. The second possibility, as you recognise, seems more sensible. I think, however, that you should start your discussion with a more detailed account of our understanding of the role of elongation factors in regulating repair and stress tolerance. There may be useful insight outside photosynthesis or cyanobacteria research too. As it stands, this paragraph reads rather weak, but I think it should be the most important of your discussion. Deal with this first and then discuss the data on and role of NDH-1.

Table 1. Add a column with the coverage/depth of the genome sequence, or the ratio of total reads with the mutation v. wildtype

Line 380. Provide the details of the bioinformatic steps used to confirm the introduced mutations using whole genome sequencing? How did you assemble the genomes, how did you search for the desired mutations?

Line 386. What's a "preculture"? I have never heard that term before.

Line 390. During these 19 weeks, did you do serial passes? How many passes did you do? Or the cultures were just simply left untouched until some growth was noted, which was only after 19 weeks? Did you have to replenish the BG11 as this dried out? Please be detailed and make sure that it reads without ambiguity.

Line 398. How many weeks did this take? For consistency.

Line 400. Explain this "gradual glucose-restriction regime" experiment in full. How many cultures, what volumes, for how many days or weeks were the cells in each condition before changing concentration?

Line 404. How did you prepare genomic DNA? What kit did you use? Or was this done by Novogene?

Please can you report on the quality of your genome sequencing and the confidence of your breseq results? What sequencing depth/coverage per genome and how much of your genomic DNA was mapped into the reference per sample?

Were there any cases of contamination? This is common. Please address this explicitely showing evidence from your genomic data. If less than 99% of the DNA mapped to the reference, then you may have contamination. If this is substantial then your interpretations may be somewhat compromised.

Line 409. What is this that you uploaded to the NCBI? Draft assemblies? How many? In your revision, please provide evidence that you have uploaded not only the draft assemblies, but also the raw data to the SRA. To be extra nice with the community, it would be fantastic if you make available online the breseq outputs too (e.g. on a zenodo account or similar).

Line 413. What volume, what OD or chlorophyll concentration? Please be detailed.

Line 417. "Gels contained ~20 µg total protein." Per well? How did you measure protein concentration?

Line 440. Detail your phycocyanin purification steps in full please.

Line 445. What type of scale did you use?

Lie 449. What volume of culture?

Line 453. Provide a reference for the methanol–chloroform–water protocol or describe it in full detail.

Line 706. Add to the table the annotation/full name of the genes.

Reviewer #2

(Remarks to the Author)
During an attempt to substitute cyanobacterial PSI with the angiosperm version, the authors discovered that some cyanobacterial strains could grow photo-autotrophically in the absence of PSI. This growth required a mutation in the fusA gene, which encodes the EF-G factor. In the mutant, it was impossible to knock out the ndhB gene, which encodes an essential subunit of the NDH-1 complex. The authors concluded that the reverse reaction of the NDH-1 complex, which is PQ-dependent Fd reduction energized by high pmf, can bypass PSI to energize CO2 fixation.

Discovery is potentially exciting. However, the authors did not test the specific electron transport, and the conclusion is not supported experimentally. In particular, I have a serious concern about the origin of pmf that promotes the verse reaction.

Specific concerns
1) The authors claimed that the NDH-1 complex's reverse reaction bypasses PSI for PSII-dependent NADP+ reduction. They also noted that the reverse reaction likely requires a high pmf. How does the mutant form the pmf in the absence of LEF and CEF? Does the line form a sufficiently high pmf in the light?
2) A level of pmf may be established when PSII activity is coupled with respiration-dependent PQ oxidation. This possibility should be tested by separately analyzing PSII-dependent O2 evolution and respiration-dependent O2 uptake.

3) To conclude the mechanism, it is necessary to quantitatively evaluate photosynthesis. Is PSII activity sufficient to explain the rate of CO_2 fixation observed?
4) It would be difficult to evaluate the reverse reaction of the NDH-1 complex. However, it is at least necessary to demonstrate the light-dependent reduction of $NADP^+$.
5) The mutation in the EF-G factor is key to photoautotrophic growth. I do not find a reasonable explanation of how the mutation contributed to the phenotype.

Version 1:

Reviewer comments:

Reviewer #1

(Remarks to the Author)
All my points have been addressed thoroughly. I have no further comments.

Reviewer #2

(Remarks to the Author)
The author adequately addressed my concerns, and I was satisfied with the revisions. In my second comment, I suggested measuring O_2 evolution and consumption simultaneously using membrane inlet mass spectrometry, for example. However, I understand that the experiment is not very simple. The quantitative discussion was supported by other approaches, so I do not request additional experiments. I like this version of the manuscript because it focuses on the mechanism rather than merely appealing to the unexpected discovery.

Open Access This Peer Review File is licensed under a Creative Commons Attribution 4.0 International License, which permits use, sharing, adaptation, distribution and reproduction in any medium or format, as long as you give appropriate credit to the original author(s) and the source, provide a link to the Creative Commons license, and indicate if changes were made.
In cases where reviewers are anonymous, credit should be given to 'Anonymous Referee' and the source.
The images or other third party material in this Peer Review File are included in the article's Creative Commons license, unless indicated otherwise in a credit line to the material. If material is not included in the article's Creative Commons license and your intended use is not permitted by statutory regulation or exceeds the permitted use, you will need to obtain permission directly from the copyright holder.
To view a copy of this license, visit https://creativecommons.org/licenses/by/4.0/

Reviewer #1 (Remarks to the Author):

Ludwiczak et al. report the serendipitous discovery of strains of Synechocystis sp. PCC 6803 capable of photosynthetic growth in the absence of photosystem I. The authors provide a full characterisation of various engineered and evolved strains using genomics and photosynthesis experiments, showing that there is a degree of photosynthetic growth in some of these strains in the absence of PSI. Restoration of photosynthetic growth is attributed to the emergence of mutations on the translation elongation factor G (FusA) and another uncharacterised protein. It is suggested that electrons flow in reverse via NDH-1 to ferredoxin to sustain carbon fixation.

I find the data presented sufficiently compelling to support the conclusion of restoration of photosynthetic growth in the absence of PSI. The role of NDH-1, while a likely explanation and a valid hypothesis, I think it is only indirectly supported with the presented data, which I find adequate at this stage.

I do not have any major concerns. Below I provide some comments aimed at improving clarity:

1.1 Lines 85-89. What is your best hypothesis for why the aiao#1 and aiao#2 strains are strictly heterotrophic despite containing a full complement of PSI genes? What about your engineering approach aimed at introducing PSI from A. thaliana means that there is no expression despite successful integration? **I think it is appropriate to address this issue in the discussion.**

Reply 1.1: To keep the Discussion section as streamlined as possible, we have decided not to open a new paragraph on this topic in the Discussion section. Instead, we addressed this issue in the Results section, immediately following the sentence describing how the lines cannot accumulate PSI. We now write: "*This finding suggests that the introduced plant PSI subunits are either insufficiently expressed or fail to assemble properly in Synechocystis, potentially due to the absence of plant-specific assembly factors that cannot be functionally replaced by endogenous components.*".

1.2 Line 89. I think some of the supplementary data/table/figures in the provided excel file requires explanatory legends. Please add to all cases in which this is missing. Do not assume that anything is self-explanatory.

Reply 1.2. We have now revised the source data and added extensive description/legends.

1.3 Line 123. "putative sugar kinase," I'm not sure this is accurate enough. Please be more accurate on how homologues of this gene are annotated beyond Synechocystis 6803.

Reply 1.3. We write now: "*The lmbP gene encodes a member of the GHMP kinase superfamily, a group of structurally related enzymes that catalyse ATP-dependent phosphorylation of small metabolites and play essential roles in pathways such as amino acid biosynthesis, carbohydrate metabolism, and the mevalonate pathway*[28, 29]*. Indeed, network interaction analyses*[30] *show LmbP clustering with metabolic enzymes, including a phosphoheptose isomerase and other proteins involved in central metabolism, supporting its proposed function in sugar metabolism or carbohydrate processing. Accordingly, it has been annotated as a "probable sugar kinase" in Synechocystis sp. LKSZ1.*"

1.4 Line 176. It is implied that the deletion of the ndhB gene in the wild-type strain does not abolish photosynthesis. Is this the case? Please state or discuss with appropriate citations.

Reply 1.4: The reviewer is correct. We write now: "We next disrupted the *ndhB* gene, essential for NDH-1 assembly[39,40] but dispensable for photoautotrophic growth in a WT background[40,41,42,43], in the *aiao-evo*$_c$ strain."

Discussion

Lines 191-195. Adding more results at the start of the discussion is confusing. There is also ambiguity and vagueness on what you report here. Please rephrase/restructure as necessary.

Reply 1.5: We mention this result now in the beginning of the Results section ("*During the third step of this replacement process, a 4.7 kb deletion upstream of psaF - encompassing sll1217, which encodes a PGRL1-like protein that functions in CEF in concert together with Pgr5*[23] *- was introduced inadvertently and subsequently restored in step 11 (**Fig. 2a***").

We shortened the corresponding passage in the beginning of the discussion section and write now: *"The extended construction and maintenance of our aiao lines under low-light conditions likely facilitated the spontaneous emergence of one of these critical mutations. Supporting this interpretation, even non-evolved aiao mutants exhibit an enhanced light-induced proton motive force (pmf) formation signature and WT-like PSII activity, whereas a newly generated ΔpsaAB mutant displays P515 kinetics similar to those of the parental WT and very little PSII activity (**Fig. 6c,f**). In addition, the transient deletion of sll1217, a component of CEF*[23]*, may have triggered compensatory adaptations influencing NDH-1 activity or alternative PSI-bypass pathways."*

1.6 Line 196. I think you should be more cautious about how you present the data and interpretations on the role of NDH-1. One could argue that your strains are already at the edge of survivability under photosynthetic conditions, and I think it is possible that any further perturbation could tip them over. Unless you measure electron transfer from PQ to Fd via NDH-1, I think you should keep this attribution as a hypothesis and toned down.

Reply 1.6: Good suggestion: We start the paragraph now with the following sentence: "Disruption of NDH-1 abolished photoautotrophic growth in the evolved strains, suggesting that the PSI-deficient strains are either operating at the very edge of photosynthetic viability - where additional perturbations such as loss of NDH-1 become lethal - or that NDH-1 functionally compensates for the absence of PSI. The elevated pmf levels observed in *aiao* #1 and its photoautotrophic derivatives (see **Fig. 6**) are consistent with NDH-1 operating in reverse, transferring electrons from PQ to Fd."

1.7. Line 217. "this unique combination of adaptive mutations…" What combination of mutations do you mean exactly?

Reply 1.7: We specify this now in the text: "… *how might adaptive mutations in fusA or lmbP, together with changes in at least one additional locus, enable NDH-1–mediated electron flow to support photoautotrophy*"

1.8. Line 219, 220. “the mutations…” be more specific, what mutations exactly?

Reply 1.8: We refer in the text now to the definition provided in Reply 1.7 and write now: “*Given that FusA contributes to oxidative stress tolerance and PSII repair*[26,27] *and LmbP is annotated as a probable sugar kinase, how might adaptive mutations in fusA or lmbP, together with changes in at least one additional locus, enable photoautotrophy in the absence of PSI?*”

1.9 Out of the two explanations you suggest, the first one does not make much sense. At least you do not provide a rationale for how this could be possible. The second possibility, as you recognise, seems more sensible. I think, however, that you should start your discussion with a more detailed account of our understanding of the role of elongation factors in regulating repair and stress tolerance. There may be useful insight outside photosynthesis or cyanobacteria research too. As it stands, this paragraph reads rather weak, but I think it should be the most important of your discussion. Deal with this first and then discuss the data on and role of NDH-1.

Reply 1.9: We follow up to our reply to 1.8 and continue the paragraph with the following sentences. “Two non-exclusive scenarios can be considered. First, this specific set of mutations could modulate the capacity of the proposed NDH-1- and oxidase-dependent electron flow pathways underpinning photoautotrophic growth. Indirect connections, such as translation elongation efficiency constraining respiratory capacity, or LmbP supporting plastoquinone biosynthesis akin to other GHMP kinases, can be envisaged, yet no such mechanisms have been demonstrated, and direct causal links remain elusive. A second explanation is that these mutations primarily mitigate the deleterious effects of PSI loss - by stabilizing PSII function, balancing cellular redox state, or improving translational regulation under stress. This interpretation aligns with the higher growth rate, increased PSII abundance and activity, and elevated oxygen evolution observed in *aiao* #1 relative to the Δ*psaAB* control (**Figs. 3** and **6**), and is consistent with the established roles of FusA in stress tolerance and PSII repair[26,27]. Moreover, adaptive mutations in the elongation factor G homologue FusB/Sll1098 have been shown to enhance high-light tolerance in *Synechocystis* by reducing antenna size[51,52], and PSI-deficient mutants of *Synechocystis* exhibit decreased photosensitivity when phycobilisomes are removed[33]. Collectively, these findings link adaptive changes in elongation factor G proteins to improved photoprotection in PSI-lacking strains.”

As suggested, we moved the tentative functions of LmbP and FusA to the beginning of the paragraph and built our argument from this foundation. The established roles of FusA and FusB indeed provide a compelling framework to interpret our findings. In the previous version, however, the adaptive effects of the mutation in FusB were likely discussed too briefly.

1.10. Table 1. Add a column with the coverage/depth of the genome sequence, or the ratio of total reads with the mutation v. wildtype

Reply 1.10: We thank the reviewer for this suggestion. To provide a clearer picture of the sequencing reliability, we have updated Table 1 to include a dedicated column for sequencing depth at each mutation site. As shown in the revised table, the depth across all identified mutations remains robust, ranging from 121x to 215x, ensuring high confidence in the called variants.

1.11. Line 380. Provide the details of the bioinformatic steps used to confirm the introduced mutations using whole genome sequencing? How did you assemble the genomes, how did you search for the desired mutations?

Reply 1.11: We appreciate the opportunity to clarify our bioinformatics workflow. As detailed in the Methods section (Lines 405-410), we performed reference-based resequencing rather than de novo assembly. Briefly, reads were trimmed using Trimmomatic and aligned to the Synechocystis PCC 6803 reference (ASM972v1) via Bowtie2. Variants were called using the Breseq (v0.39.0) pipeline. To ensure high confidence in our results, we only considered mutations with a sequencing depth exceeding 100x. Furthermore, the parental strain sequence was used as a baseline to subtract background mutations, ensuring that only newly introduced mutations in the evolved strains were reported. Large structural variations and deletions were specifically screened using Breseq's junction-prediction algorithm.

A corresponding passage has been inserted now in the Methods section: "To identify the potential mutations of interest, the sequence of the parental strain was used as a baseline from which to subtract background mutations. Only mutations with more than 100x coverage were considered. Large structural variations and deletions were screened using the junction-prediction algorithm in Breseq (v0.39.0)[52]."

We would like to point out that we didn't perform a de-novo assembly of our genome. We employed a reference-based resequencing pipeline using breseq.

1.12. Line 386. What's a "preculture"? I have never heard that term before.

We use the term "preculture" in accordance with previous publications such as Wang et al., 2024. Appl Microbiol Biotechnol. 108(1):117. In this context, "preculture" refers to a preliminary cultivation step where our cyanobacteria (commonly maintained on BG11 agar media) are grown in advance of the main experiment to acclimate them to the liquid BG11 media conditions, thus synchronizing/reducing the lag phase upon transition into the main experimental culture.

We write now: " 'precultures' and introduced the explanation: "This preliminary cultivation step, carried out prior to the main experiment, served to acclimatize the cultures to the liquid BG11 medium."

1.13. Line 390. During these 19 weeks, did you do serial passes? How many passes did you do? Or the cultures were just simply left untouched until some growth was noted, which was only after 19 weeks? Did you have to replenish the BG11 as this dried out? Please be detailed and make sure that it reads without ambiguity.

Reply 1.13. We now specify in the methods section that "During this period, no additives were provided and the BG11 media was not replenished, with volume loss due to evaporation being minimal due to aluminium foil sealing of the culture flasks."
Part of such aluminium foil capping minimizing evaporation can be seen in Supplementary Fig. 2, bottom left.

1.14. Line 398. How many weeks did this take? For consistency.

Reply 1.14: We added now “after 15 weeks”

1.15. Line 400. Explain this “gradual glucose-restriction regime” experiment in full. How many cultures, what volumes, for how many days or weeks were the cells in each condition before changing concentration?

Reply 1.15: We added a whole paragraph detailing the 'gradual glucose-restriction regime'.

1.16. Line 404. How did you prepare genomic DNA? What kit did you use? Or was this done by Novogene?

Reply 1.16: We have added: “using the EasyPure Plant Genomic DNA Kit (TransGen Biotech)”

1.17. Please can you report on the quality of your genome sequencing and the confidence of your breseq results? What sequencing depth/coverage per genome and how much of your genomic DNA was mapped into the reference per sample?

Reply 1.17: We have provided additional metrics to demonstrate the high quality of our whole-genome sequencing and the resulting reliability of the breseq analysis. As shown in the newly added **Supplementary Table 2**, the sequencing depth remained high across all genomic elements, with chromosomal coverage ranging from 116.1x to 310.4x. Furthermore, the alignment quality was excellent, with 97.96% to 99.49% of all reads mapping successfully to the *Synechocystis* PCC 6803 reference genome. The percentage of mapped reads ranged from 97.96% to 99.49% (**Supplementary Table 3**).

1.18. Were there any cases of contamination? This is common. Please address this explicitely showing evidence from your genomic data. If less than 99% of the DNA mapped to the reference, then you may have contamination. If this is substantial then your interpretations may be somewhat compromised.

Reply 1.18: We thank the reviewer for this important check on data quality. We have explicitly analyzed the mapping statistics and potential for contamination across all samples.

While the alignment rates for our samples ranged from 97.96% to 99.49% (see Reply 1.17), we do not find evidence of biological contamination that would compromise our interpretations. In microbial evolution experiments, alignment rates slightly below 99% are common and often reflect structural variations or the presence of highly repetitive sequences that breseq might filter out to maintain high specificity (Deatherage & Barrick, 2014).

Deatherage, D. E. & Barrick, J. E. Identification of mutations in laboratory-evolved microbes from next-generation sequencing data using breseq. Methods Mol Biol 1151, 165-188 (2014). https://doi.org/10.1007/978-1-4939-0554-6_12

To address this explicitly, we performed the following:

1. Taxonomic screening: We extracted all unmapped reads from the sample with the lowest alignment rate (aiao #1, 97.96%) and analyzed them using Kraken2. The analysis showed that

>99% of these reads were either "unclassified" (likely due to low complexity or sequencing artifacts) or matched the genus of our target organism, with no significant presence (<1%) of foreign DNA (see the figure below). **<u>The full output report of Kraken2 is attached.</u>**

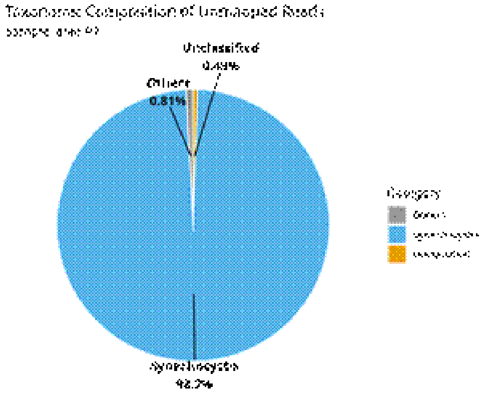

2. Coverage depth: For all sequenced strains, the coverage was always larger than 150x. Even with ~2% unmapped reads, the breadth of coverage across the reference genome remained higher than 147%, ensuring that our mutation calls are supported by robust local evidence.

Consequently, we are confident that the small fraction of unmapped reads does not represent contamination and does not impact the validity of the identified mutations.

1.19. Line 409. What is this that you uploaded to the NCBI? Draft assemblies? How many? In your revision, please provide evidence that you have uploaded not only the draft assemblies, but also the raw data to the SRA. To be extra nice with the community, it would be fantastic if you make available online the breseq outputs too (e.g. on a zenodo account or similar).

Reply 1.19: We thank the reviewer for the opportunity to clarify our data deposition. We have uploaded the raw sequencing reads (FASTQ files) for all strains (including both forward and reverse reads) to the NCBI Sequence Read Archive (SRA) under BioProject PRJNA1380123. These can be accessed via the provided reviewer link.

https://dataview.ncbi.nlm.nih.gov/object/PRJNA1380123?reviewer=372a9hs11f9f4521c95mo5qr6m

Furthermore, to support community reuse and full transparency, we have followed the reviewer's suggestion and uploaded the complete breseq output files (.gd) along with the indexed alignment files (.bam and .bai) to a Zenodo repository (https://doi.org/10.5281/zenodo.18668074). These records provide the necessary data for independent validation of our variant calls and will be made publicly available immediately upon the publication of this manuscript.

1.20. Line 413. What volume, what OD or chlorophyll concentration? Please be detailed.

Reply 1.20: We write now: "Aliquots of 30 ml of cultures (OD_{720} = 1) of cells were ...."

1.21. Line 417. "Gels contained ~20 µg total protein." Per well? How did you measure protein concentration?

Reply 1.21: We write now: "Gels contained ~20 µg total protein per lane" and "… the protein concentration was determined using the BCA assay (Thermo Fisher Scientific, MA, USA),…"

1.22. Line 440. Detail your phycocyanin purification steps in full please.

Reply 1.22: We provide now details: Phycocyanin was isolated after cell disruption in PBS (pH 7.4) with glass beads using a TissueLyserII (Qiagen) at 30 Hz for 5 min. The lysate was then centrifuged twice at 4 °C, first at 4000 g for 5 min, and subsequently at 16000 g for 5 min using the collected supernatant. The absorbance of the resulting supernatant was measured at 615 and 652 nm[59], and pigment concentrations were determined using standard calculation formulas.

1.23. Line 445. What type of scale did you use?

Reply 1.23: We add now: "… using an analytical balance (KERN, Germany)."

1.24. Lie 449. What volume of culture?

Reply 1.24: We write now: "100 ml of each culture were collected, the glucose washed out, and the cells were ..."

1.25. Line 453. Provide a reference for the methanol–chloroform–water protocol or describe it in full detail.

Reply 1.25: The reference was provided.

1.26. Line 706. Add to the table the annotation/full name of the genes.

Reply 1.26: done

Reviewer #2 (Remarks to the Author):

2.0 During an attempt to substitute cyanobacterial PSI with the angiosperm version, the authors discovered that some cyanobacterial strains could grow photo-autotrophically in the absence of PSI. This growth required a mutation in the fusA gene, which encodes the EF-G factor. In the mutant, it was impossible to knock out the ndhB gene, which encodes an essential subunit of the NDH-1 complex. The authors concluded that the reverse reaction of the NDH-1 complex, which is PQ-dependent Fd reduction energized by high pmf, can bypass PSI to energize CO2 fixation.

Discovery is potentially exciting. However, the authors did not test the specific electron transport, and the conclusion is not supported experimentally. In particular, I have a serious concern about the origin of pmf that promotes the verse reaction.

Reply 2.0: See Replies 2.1 and 2.2

Specific concerns
1) The authors claimed that the NDH-1 complex's reverse reaction bypasses PSI for PSII-dependent NADP+ reduction. They also noted that the reverse reaction likely requires a high pmf. How does the mutant form the pmf in the absence of LEF and CEF? Does the line form a sufficiently high pmf in the light?

Reply 2.1: Using a recently established measurement routine based on long-term P515 signal accumulation under actinic light treatment (Zhivkovikj et al., Physiologia Plantarum, 177(3)), we observed indication of enhanced pmf formation in all PSI-deficient mutants with the exception of a *psaAB* deletion strain. This corroborates the notion of a relatively strong pmf being formed in absence of canonical LEF and points towards enabling mutation being acquired by aiao prior to directed ALE for photoautotropy.

We write in the Results section: “All PSI-deficient strains, with the exception of Δ*psaAB*, showed enhanced proton motive force (pmf) formation upon illumination with actinic light (**Fig. 6e**). Using a recently established non-invasive P515 measurement approach[34], we observed rapid light-induced pmf generation and elevated steady-state pmf levels in *aiao*#1 and its evolved derivative strains.”

We write in the discussion section: “Supporting this interpretation, even non-evolved *aiao* mutants exhibit an enhanced light-induced proton motive force (pmf) formation signature, whereas a newly generated Δ*psaAB* mutant displays P515 kinetics similar to those of the parental WT (**Fig. 6e**)”

2) A level of pmf may be established when PSII activity is coupled with respiration-dependent PQ oxidation. This possibility should be tested by separately analyzing PSII-dependent O2 evolution and respiration-dependent O2 uptake.

Reply 2.2: We provide now data on PSII activity and respiration measurements in the new Figure 6. We write now in the Results section: “In fact, direct measurements of PSII activity, based on O_2 evolution in the presence of DCBQ and $K_3Fe(CN)_6$, revealed higher values in the *aiao* #1 strain relative to Δ*psaAB*. Meanwhile, PSI-deficient photoautrophic strains retained WT-like PSII activity, with the exception of *aiao-evo*$_{ext}$, which showed reduced rates (**Fig. 6c**). Respiration rates, calculated from the data underlying Fig. 6b, were comparable across all genotypes (**Fig. 6d**).”

3) To conclude the mechanism, it is necessary to quantitatively evaluate photosynthesis. Is PSII activity sufficient to explain the rate of CO2 fixation observed?

Reply 2.3: All evolved strains clearly incorporate CO_2, as demonstrated by the uptake of ^{13}C-labelled bicarbonate and the corresponding isotopic enrichment detected in sucrose (Fig. 6a). To assess whether PSII activity supplies sufficient electrons to support CO_2 fixation and net biomass accumulation (dry mass), we compared photoautotrophic growth (Fig. 5c) with net O_2 evolution rates (Fig. 6b). Relative to the wild type (WT), all evolved PSI-deficient mutants exhibit a slightly higher ratio of PSII-derived electron flux (reflected by net O_2 evolution) to biomass accumulation. This indicates that PSII provides an adequate electron supply to reduce the CO_2 required for the observed biomass production in evolved PSI-free strains. The modest reduction in growth efficiency may result from the diminished contribution of NDH-1 to the cellular carbon-concentrating mechanism (CCM) when electron flow through NDH-1 operates in reverse.

We write in the discussion section. “Consistent with this framework, evolved PSI-deficient strains exhibit a modestly disproportionate reduction in biomass accumulation relative to net O_2 evolution. On average, dry mass accumulation reaches 44 ± 2% (*aiao-evo*$_c$), 37 ± 7% (*aiao-evo*$_{ext}$), and 42 ± 5% (ΔPSI*) of WT levels, whereas net O_2 evolution attains 29 ± 2%, 29 ± 6%, and 24 ± 16% of WT, respectively (**Fig. 5c**; **Fig. 6b**). This reduced biomass yield per unit of photosynthetic activity may reflect partial uncoupling within the proposed energetic network, potentially compounded by the

operation of proton-removal mechanisms associated with NDH-$1_{3/4}$ isoforms that exhibit carbonic anhydrase activity and support cellular carbon fixation[50]."

Moreover, the net O_2 evolution in the PSI-deficient strains of between 43% and 47% of WT aligns very well with the predicted net oxygen evolution that we predicted based on our electron partitions calculations (new paragraph at the end of the Results section) in which we write: "Thus, at a fixed Calvin–Benson ATP/NADPH ratio, the Cox-only mode generates the highest pmf and ATP yield per $2e^-$, while sustaining slightly more than 50% of the net oxygen production observed in WT cells operating LEF and CEF with PSI. However, if an elevated pmf were to inhibit Cyt b_6f activity[44], thereby limiting electron supply to Cox and shifting O_2 reduction predominantly to Cyd, net O_2 evolution would decline to approximately 30% of WT levels (**Table 3**)."

As noted in the previous version, PSII abundance is elevated in the evolved strains. We now further show that PSII activity (see new Fig. 6) is WT-like in aiao #1 and most of its derived autotrophic derivatives. We write: "*In fact, direct measurements of PSII activity, based on O_2 evolution in the presence of DCBQ and $K_3Fe(CN)_6$, revealed higher values in the aiao #1 strain relative to ΔpsaAB. Meanwhile, PSI-deficient photoautrophic strains retained WT-like PSII activity, with the exception of aiao-evo$_{ext}$, which showed reduced rates (**Fig. 6c**).*" Taken together, these findings indicate that PSII capacity persists in the evolved lines. We therefore consider the question of whether PSII activity is sufficient to sustain photosynthetic growth to be addressed both theoretically—through modelling of electron partitioning—and experimentally—through the relationship between net O_2 evolution and biomass accumulation.

4) It would be difficult to evaluate the reverse reaction of the NDH-1 complex. However, it is at least necessary to demonstrate the light-dependent reduction of NADP+.

Reply 2.4: Actually, measuring NADPH generation is also difficult and we had to establish this in our laboratory. The results are now presented in Fig. 6e. We write in the results section: "The kinetics of light-induced NADPH formation and its subsequent dark consumption were assessed in vivo by monitoring NADPH fluorescence[34,35]. Upon illumination, the amplitude of the NADPH signal was elevated in the *aiao* #1, *aiao-evo*$_c$ and DPSI* strains relative to WT and Δ*psaAB* (**Fig. 6e**), indicating enhanced NADPH formation, a reduced or delayed rate of NADPH-consuming reactions in the light, or a more oxidised NADPH pool in the dark in *aiao* #1 and *aiao-evo*$_c$. In contrast, the *aiao-evo*$_{ext}$ strain exhibited a lower NADPH signal amplitude."

5) The mutation in the EF-G factor is key to photoautotrophic growth. I do not find a reasonable explanation of how the mutation contributed to the phenotype.

Reply 2.5. We had addressed this in the previous version but too short. We write now: "Given that LmbP is annotated as a probable sugar kinase and FusA contributes to oxidative stress tolerance and PSII repair[26,27], how might adaptive mutations in *fusA* or *lmbP*, together with changes in at least one additional locus, enable photoautotrophy in the absence of PSI? Two non-exclusive scenarios can be considered. First, this specific set of mutations could modulate the capacity of the proposed NDH-1- and oxidase-dependent electron flow pathways underpinning photoautotrophic growth. Indirect connections, such as translation elongation efficiency constraining respiratory capacity, or LmbP supporting plastoquinone biosynthesis akin to other GHMP kinases, can be envisaged, yet no such mechanisms have been demonstrated, and direct causal links remain elusive. A second explanation is

that these mutations primarily mitigate the deleterious effects of PSI loss - by stabilizing PSII function, balancing cellular redox state, or improving translational regulation under stress. This interpretation aligns with the higher growth rate, increased PSII abundance, and elevated oxygen evolution observed in *aiao #1* relative to the Δ*psaAB* control (Figs. 2–4), and is consistent with the established roles of FusA in stress tolerance and PSII repair[26,27]. Moreover, adaptive mutations in the elongation factor G homologue FusB/Sll1098 have been shown to enhance high-light tolerance in *Synechocystis* by reducing antenna size[49,50], and PSI-deficient mutants of *Synechocystis* exhibit decreased photosensitivity when phycobilisomes are removed[33]. Collectively, these findings link adaptive changes in elongation factor G proteins to improved photoprotection in PSI-lacking strains."
